# RNAi-based ALOX15B silencing augments keratinocyte inflammation in vitro via EGFR/STAT1/JAK1 signalling

**DOI:** 10.1038/s41419-025-07357-x

**Published:** 2025-01-22

**Authors:** Megan A. Palmer, Rebecca Kirchhoff, Claudia Buerger, Yvonne Benatzy, Nils Helge Schebb, Bernhard Brüne

**Affiliations:** 1 Faculty of Medicine, Institute of Biochemistry I, Goethe University Frankfurt, Frankfurt, Germany; 2 Chair of Food Chemistry, School of Mathematics and Natural Sciences, University of Wuppertal, Wuppertal, Germany; 3 Department of Dermatology, Venerology and Allergology, Goethe University Frankfurt, University Hospital, Frankfurt am Main, Germany; 4 Fraunhofer Institute for Translational Medicine and Pharmacology ITMP, Frankfurt, Germany; 5 German Cancer Consortium (DKTK), Partner Site Frankfurt, Frankfurt, Germany

**Keywords:** Mechanisms of disease, Inflammation, Interferons

## Abstract

Arachidonate 15-lipoxygenase type B (ALOX15B) peroxidises polyunsaturated fatty acids to their corresponding fatty acid hydroperoxides, which are subsequently reduced into hydroxy-fatty acids. A dysregulated abundance of these biological lipid mediators has been reported in the skin and blood of psoriatic compared to healthy individuals. RNAscope and immunohistochemistry revealed increased ALOX15B expression in lesional psoriasis samples. Using a cytokine cocktail containing IL-17A, interferon-gamma and tumour necrosis factor-alpha to produce a psoriasis-like phenotype, a role for ALOX15B in human epidermal keratinocyte inflammation was investigated. siRNA-mediated silencing of ALOX15B increased CCL2 expression and secretion. In addition to CCL2, secretion of CCL5 and CXCL10 were elevated in skin equivalents treated with lipoxygenase inhibitor ML351. Inhibition of the JAK1/STAT1 pathway reversed the enhanced CCL2 expression found with ALOX15B silencing. Previous studies have linked epidermal growth factor receptor (EGFR) inhibition with the upregulation of cytokines including CCL2, CCL5 and CXCL10. ALOX15B silencing reduced EGFR expression and inhibition of EGFR signalling potentiated the effect of ALOX15B silencing on increased CCL2, CCL5 and CXCL10 expression. Confirming previous findings, gene expression of cholesterol biosynthesis genes was reduced via reduced ERK phosphorylation. Reduced ERK phosphorylation was dependant on EGFR and NRF2 activation. Furthermore, plasma membrane lipids were investigated via confocal microscopy, revealing reduced cholesterol and lipid rafts. This study suggests a role for ALOX15B in keratinocyte inflammation through modulation of lipid peroxidation and the EGFR/JAK1/STAT1 signalling axis.

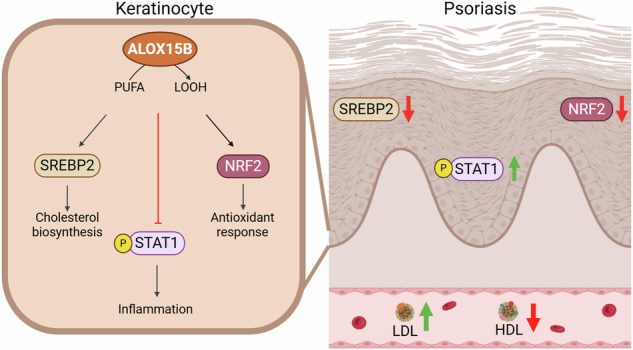

## Introduction

Lipids are essential in cutaneous biology, which is underscored by their importance in the formation of the permeability barrier as well as their involvement in modulating inflammation [1], cell growth and differentiation [2]. Psoriasis is a chronic inflammatory skin disease affecting approximately 3% of the population worldwide [3]. Characterised by hyperproliferation and abnormal differentiation of keratinocytes along with the infiltration of immune cells into the epidermis, leading to thickening skin with scales [4]. Moreover, dysregulation in the abundance of biological lipid mediators has been reported in psoriatic skin and blood compared to healthy individuals [5].

Lipoxygenases (LOX) are a family of enzymes that peroxidise polyunsaturated fatty acids (PUFA) to their corresponding fatty acid hydroperoxides [6]. These short-lived hydroperoxides are subsequently reduced into pro- or anti-inflammatory hydroxy-fatty acids. There are six functional human LOX enzymes, named for the corresponding carbon of arachidonic acid (AA) in which molecular oxygen is inserted. In the skin, arachidonate 5-lipoxygenase (ALOX5) expression is restricted to Langerhans’s [7] and infiltrating immune cells along with some fibroblasts [8]. A dual role for ALOX12B and ALOXE3 has been described in the formation of ω-hydroxyceramides, an essential component of the cornified lipid envelope [9]. Of the two 15-LOX, only ALOX15B is expressed in normal human skin [10].

ALOX15B has been associated with psoriasis, with increases in both the expression of ALOX15B [11, 12] and elevation of its AA, linoleic acid and docosahexaenoic acid (DHA) metabolites, 15-hydroxyeicosatetraenoic acid (HETE) [13] 13-hydroxyoctadecadienoic acid and 17-hydroxydocosahexaenoic acid, respectively [14]. Moreover, intralesional injection of 15-HETE in patients with psoriasis was shown to aid resolution [15]. However, the molecular mechanism and extent to which ALOX15B may help in the resolution of psoriasis remains unknown. To further understand the biological role of ALOX15B in keratinocyte inflammation, this study employed siRNA-mediated silencing of ALOX15B in keratinocytes.

## Results

### ALOX15B is upregulated in Psoriasis

**Fig. 1:**
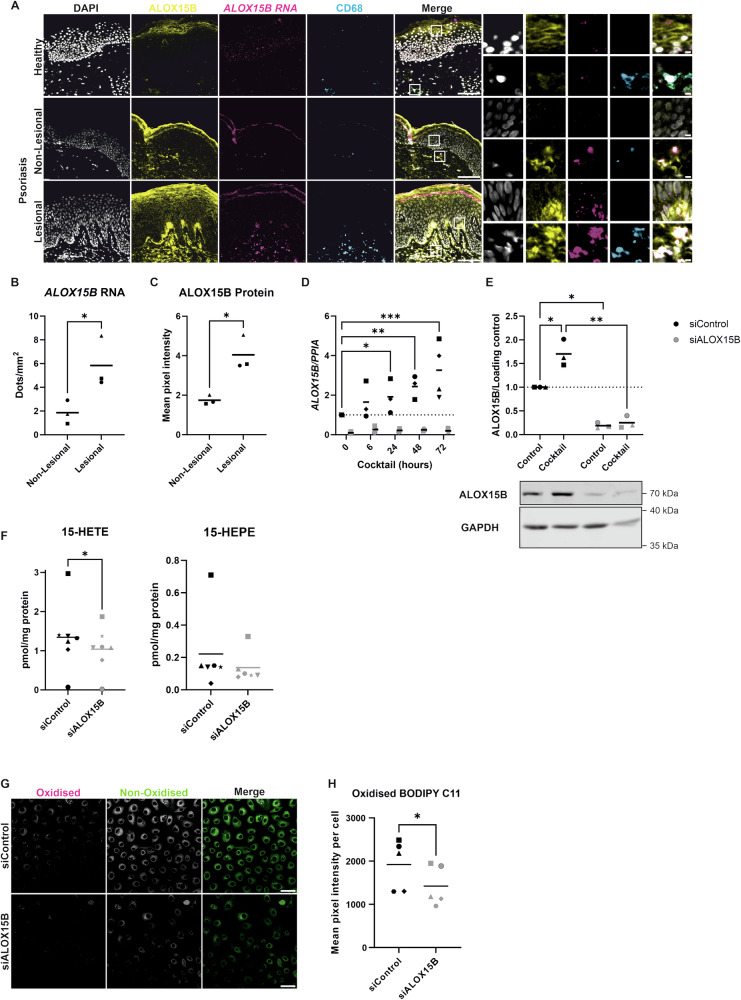
ALOX15B is upregulated in Psoriasis. **A** Representative images from dual immunofluorescence and RNAscope in human skin for ALOX15B (yellow, protein; magenta, RNA) and CD68 (cyan, macrophages). Scale bar 100 µm, white boxes represent zoomed-in regions, zoomed in region scale bar 5 µm. Analysis of (**B**) RNAscope and (**C**) immunofluorescence staining of ALOX15B, data represented as mean dots/intensity per mm^2^ of the epidermis (*N* = 3). Gene expression (**D**) or Western (**E**) analysis of ALOX15B in keratinocytes treated with control or ALOX15B siRNA for 72 h, followed by cytokine cocktail of IL-17A, IFNγ and TNFα for 6, 24, 48 or 72 h (Western blot 72 h). Gene expression is relative to control siRNA and normalised to *PPIA* (*N* = 3, or 4 for 72 h). Western analysis is relative to control siRNA and normalised to loading control (*N* = 3). Analysis of 15-LOX specific oxylipins (**F**, *N* = 7) and confocal microscopy of lipid peroxidation sensor BODIPY C11 (**G**, *N* = 5) in keratinocytes treated with control or ALOX15B siRNA for 72 h. Scale bar 50 µm, image analysis (**H**) of mean pixel intensity per cell of oxidised BODIPY C11. Individual biological replicates represented by different symbol sets, line is mean, dotted line represents normalisation to siControl at 1. *t* tests (tissue) and two-way ANOVA (cells) were performed; significance denoted by *P < 0.05, **P < 0.01, ***P < 0.001.

Firstly, the location of ALOX15B in human skin was examined via combined RNAscope and immunohistochemistry (Fig. 1A). In the epidermis, *ALOX15B* RNA was localised to the basal and suprabasal keratinocyte layers, whereas protein was expressed throughout the epidermis. An increase in intensity was detected in the more differentiated layers of the epidermis, along with plasma membrane localisation. This data coincides with primary normal human epidermal keratinocytes cultured in vitro under the presence of Ca^2+^, where an increase in ALOX15B protein expression was confirmed via Western analysis (Fig. S1A). Furthermore, immunocytochemistry staining in differentiated keratinocytes showed increased expression of ALOX15B at the plasma membrane (Fig. S1B). Next, expression of ALOX15B was analysed in non-lesional and lesional skin of psoriatic patients. Both, RNA (Fig. 1B) and protein (Fig. 1C) were elevated in the lesional epidermis samples. In addition, ALOX15B expression in the dermal compartment was restricted to CD68+ macrophages (Fig. 1A).

Given that the expression and location of ALOX15B in immortalised keratinocyte cell line NHEK/SVTERT3-5 is representative of that of primary keratinocytes (Fig. S1B), subsequent experiments were performed using NHEK/SVTERT3-5 cells. To investigate the role of ALOX15B in psoriasis, keratinocytes in vitro were treated with a cocktail of IL-17A, interferon (IFN)γ and tumour necrosis factor (TNF)α to induce a psoriasis-like phenotype following 72 h of transfection with control or ALOX15B siRNA. In control cells, *ALOX15B* expression increased over time, with significant increases detected at 24, 48 and 72 h post-treatment (Fig. 1D). Western analysis confirmed a significant increase in protein expression in control transfected cells followed by 72 h of cocktail treatment and a significant reduction in protein in ALOX15B silenced cells (Fig. 1E).

### ALOX15B silencing reduces arachidonic and eicosapentaenoic acid derived oxylipins i.e 15-HETE and 15-HEPE

To determine if ALOX15B is catalytically active under basal conditions in keratinocytes, oxylipins were quantified by LC-MS/MS. AA metabolite 15-HETE was significantly reduced and eicosapentaenoic acid (EPA)-derived 15-hydroxyeicosapentaenoic acid (HEPE) showed a trend towards lower concentrations after transfection with ALOX15B siRNA (Fig. 1F). Silencing of ALOX15B did not result in alteration of other hydroxy-fatty acids, except for 9-HEPE (Fig. S2 A-D). Relative gene expression of other members of the lipoxygenase family in keratinocytes was low compared to *ALOX15B* and was unchanged with ALOX15B silencing (Fig. S2E). Furthermore, BODIPY C11 was utilised to detect changes in lipid peroxidation (Fig. 1G), silencing of ALOX15B significantly reduced lipid peroxidation (Fig. 1H).

### ALOX15B silencing augments keratinocyte inflammation

**Fig. 2:**
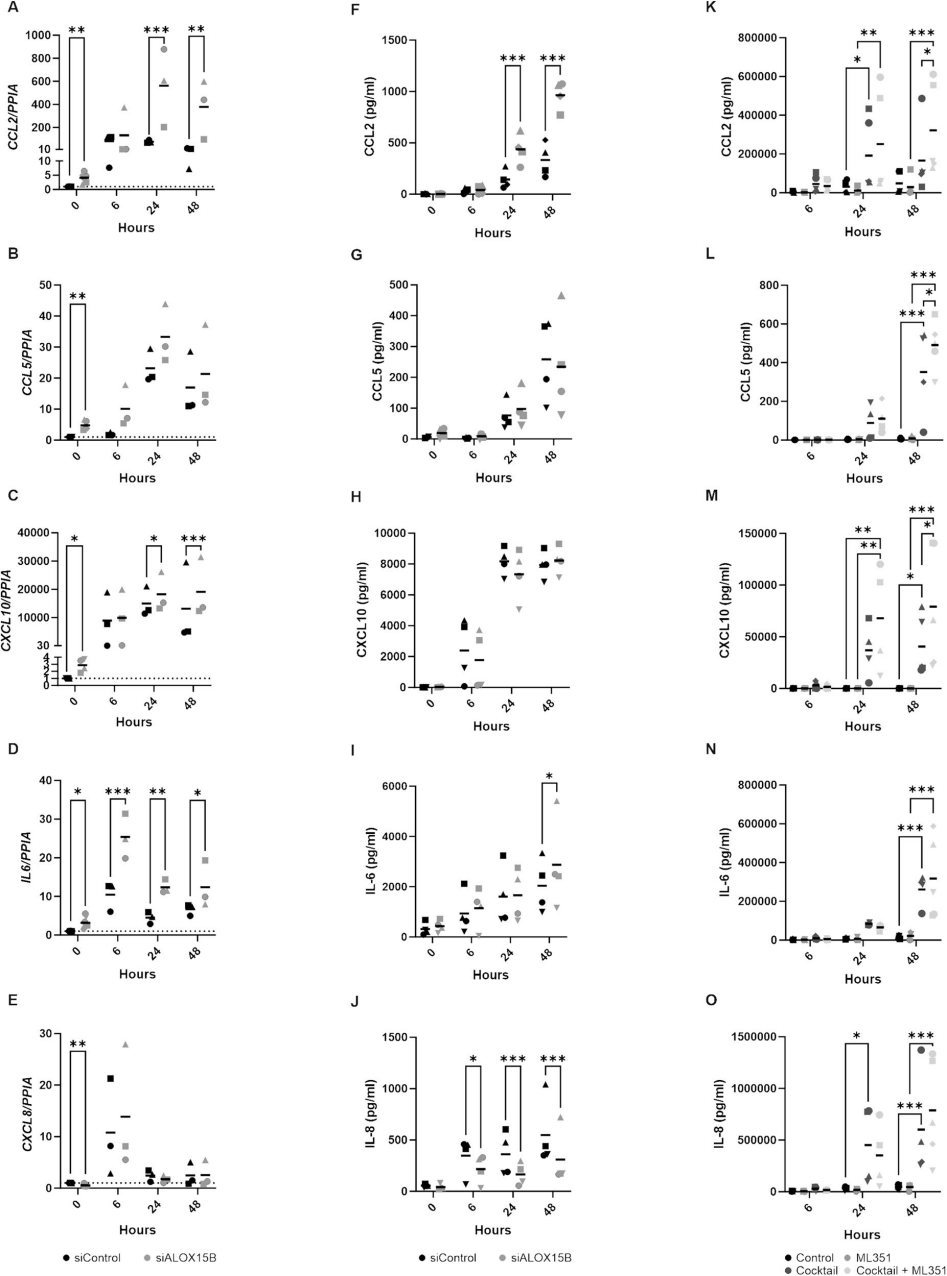
ALOX15B silencing augments keratinocyte inflammation. Gene expression (**A**–**E**: *N* = 6 for 0 h and *N* = 3 for 6–48 h) and cytokine secretion (**F**–**J**: *N* = 4) in keratinocytes transfected with control or ALOX15B siRNA for 72 h, followed by a cocktail of 50 ng/ml IL-17A, TNFα and 10 ng/ml IFNγ for 6, 24 or 48 h. Gene expression is relative to control siRNA (represented by dotted line) and normalised to *PPIA*. Cytokine secretion from human skin equivalents (**K**–**O**) treated with cytokine cocktail with or without 10 µM ML351 for 6, 24 or 48 h (*N* = 5). Individual biological replicates represented by different symbol sets, line is mean. Two-way ANOVA performed; significance denoted by *P < 0.05, **P < 0.01, ***P < 0.001.

Next, gene expression of key cytokines produced by inflammatory keratinocytes was assessed. *CCL2, CCL5, CXCL10*, *IL6* and *CXCL8* increased following cocktail treatment (Fig. 2A–E). ALOX15B silencing alone significantly increased the expression of *CCL2, CCL5, CXCL10* and *IL6*, whereas *CXCL8* was decreased. Silencing of ALOX15B significantly elevated the expression of *CCL2, CXCL10* and *IL6* following cocktail treatment in comparison to control siRNA-treated cells. Subsequently, the secretion of cytokines was analysed by cytometric bead array (Fig. 2F–J). Significant elevation of CCL2 secretion was detected following ALOX15B silencing at 24 and 48 h (Fig. 2F), and IL-6 at 48 h (Fig. 2I), whilst secretion of IL-8 was reduced from 6–48 h (Fig. 2J).

Keratinocytes cultured at the air-liquid interphase with the presence of Ca^2+^ can be used to create a more physiologically relevant 3D skin model. In addition to siRNA-mediated silencing, ML351, a lipoxygenase inhibitor, was utilised to investigate the role of ALOX15B in keratinocyte inflammation in a 3D environment. Human skin equivalents were generated using dermal fibroblast seeded into alvetex scaffolds, with keratinocytes grown at air-liquid interphase. Morphology of the skin equivalents was assessed with haematoxylin and eosin staining. Treatment with ML351 resulted in no change in epidermal thickness. Cocktail treatment provoked thickening of the epidermis and enhanced differentiation (Fig. S3A). As with 2D cell culture, secretion of CCL2, CCL5, CXCL10, IL-6 and IL-8 were elevated with the cytokine cocktail treatment after 24 or 48 h (Fig. 2K–O). Furthermore, treatment with ML351 significantly elevated CCL2, CCL5 and CXCL10 secretion (Fig. 2K–M).

ML351 treatment in 2D keratinocyte cultures reduced oxidation of BODIPY C11 (Fig. S4A) suggesting ML351 can limit lipid peroxidation in keratinocytes. Next, oxylipin measurements were performed on keratinocytes treated with ML351 for 24 h. ML351 significantly reduced many metabolites of arachidonic and linoleic acid (Fig. S4B). In addition to 15-LOX, ML351 has been reported to inhibit ALOX5 [16], therefore Western analysis of lipoxygenase and cyclooxygenase enzymes was performed (Fig. S4C). Utilising M1 polarised macrophages as a positive control for ALOX15, expression was not detected in either keratinocytes or fibroblasts. ALOX12 was detected in fibroblasts but not keratinocytes, as confirmed by positive expression in platelets. ALOX5 is known to be expressed in macrophages and neutrophils, and a faint band was present for ALOX5 for both keratinocytes and fibroblasts. Collectively these data indicate non-enzymatic reduction in lipid peroxides from ML351 treatment.

### Inhibition of JAK1/STAT1 pathway attenuates ALOX15B silencing mediated CCL2 expression

**Fig. 3:**
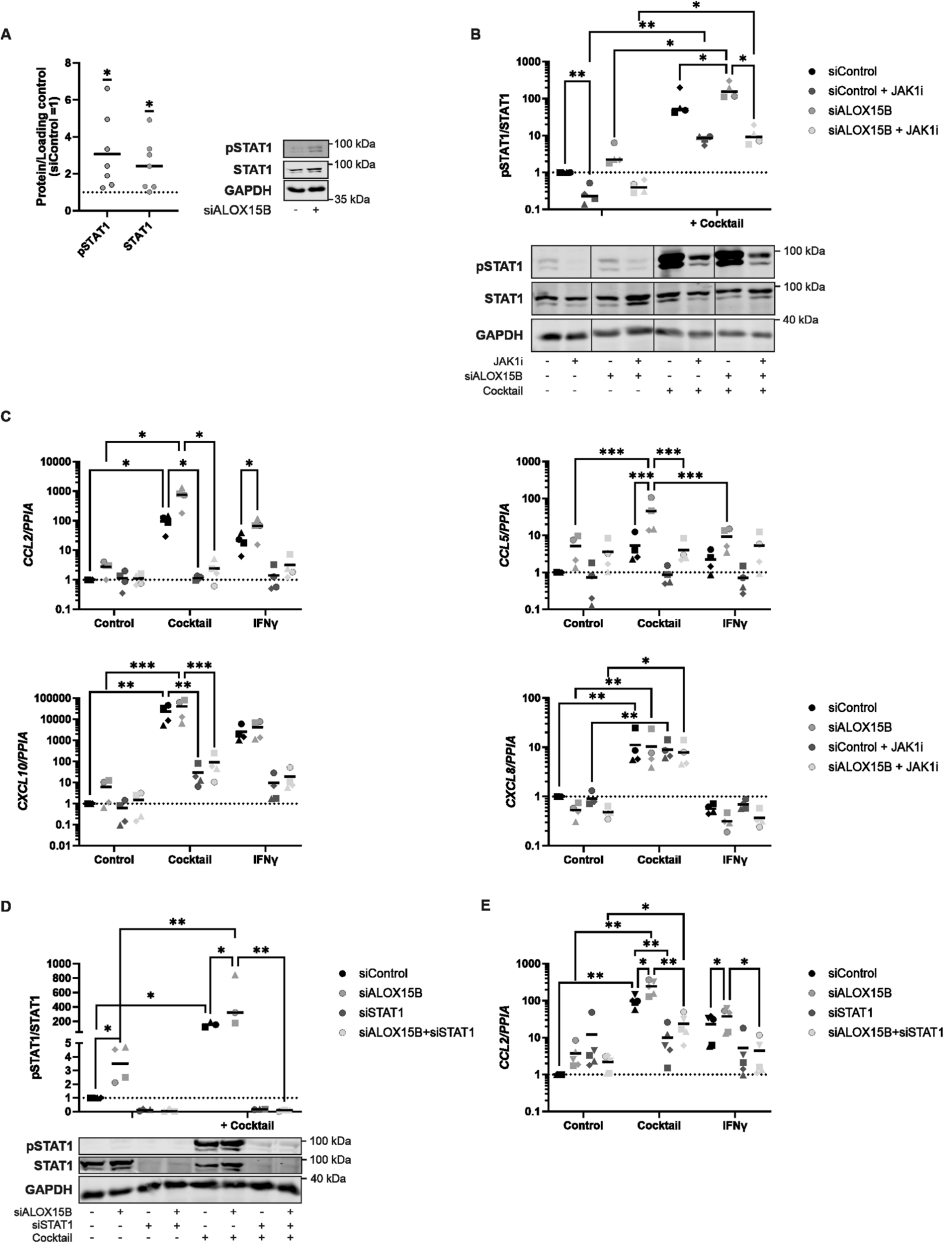
JAK1/STAT1 pathway is altered with ALOX15B silencing. **A**–**C** Keratinocytes transfected with control or ALOX15B siRNA for 72 h, (**B**, **C**) followed by 30-min pre-treatment with 1 µM JAK inhibitor I, then a cocktail of 50 ng/ml IL-17A, TNFα and 10 ng/ml IFNγ or IFNγ alone for 6 h. **D**, **E** Keratinocytes transfected with control, ALOX15B or STAT1 siRNA for 72 h, followed by cocktail treatment. Gene expression (**C**, **E**) is relative to control siRNA and normalised to *PPIA* (**C**: *N* = 4, **E**: *N* = 5). Western analysis (**A**: *N* = 6, **B**: *N* = 4, D: *N* = 4 or 3 for cocktail treated) of total and phospho STAT1 (Y701) relative to control siRNA. Individual biological replicates represented by different symbol sets, line is mean, dotted line represents normalisation to siControl at 1. Two-way ANOVA or one sample *t* test (**A**) performed; significance denoted by *P < 0.05, **P < 0.01, ***P < 0.001.

To investigate the mechanism of how ALOX15B silencing augments keratinocyte inflammation, keratinocytes were treated with the individual components of the cytokine cocktail (Fig. S5). In addition, IL-6 treatment was also performed, due to increased IL-6 expression detected upon ALOX15B silencing (Fig.2D, I). Both TNFα and IFNγ treatments alone increased *CCL2* expression, however, ALOX15B silencing significantly elevated *CCL2* following IFNγ treatment. As these data indicate altered IFNγ signalling in ALOX15B silenced keratinocytes, phosphorylation of STAT1 was assessed. Elevated phosphorylation of STAT1 in comparison to total STAT1 was observed with ALOX15B silencing, along with elevated total STAT1 in comparison to total protein (Fig. 3A).

Next, the effects of JAK1 inhibition in ALOX15B silenced keratinocytes were investigated. Inhibition of JAK1 reduced phosphorylation of STAT1 in both unstimulated and cocktail-treated cells (Fig. 3B). Furthermore, JAK1 inhibition attenuated the effect of ALOX15B silencing on STAT1 phosphorylation, revealing no differences between cocktail-treated control and ALOX15B silenced cells. To further confirm the link between JAK/STAT signalling and ALOX15B silencing, gene expression of *CCL2, CCL5, CXCL10* and *CXCL8* were analysed. Inhibition of JAK1 reduced *CCL2* expression towards basal levels in both control and ALOX15B siRNA transfected cells following treatments with either the cytokine cocktail or IFNγ alone (Fig. 3C). In addition, significant reductions in *CCL5* and *CXCL10* expression were observed (Fig. 3C).

As JAK1 interacts with many tyrosine kinase receptors and STATs, experiments with siRNA for STAT1 and ALOX15B were performed. Silencing of STAT1 with or without ALOX15B siRNA significantly reduced both total and phosphorylation of STAT1 (Fig. 3D). Furthermore, dual siRNA transfection of STAT1 and ALOX15B reduced *CCL2* expression in keratinocytes treated with either the cocktail or IFNγ alone in comparison to ALOX15B silenced keratinocytes (Fig. 3E). Collectively these data imply a role of ALOX15B in modulation of the JAK1/STAT1 pathway and confirm the involvement of JAK1/STAT1 in the transcriptional regulation of *CCL2, CCL5* and *CXCL10*.

### ALOX15B silencing inhibits EGFR

**Fig. 4:**
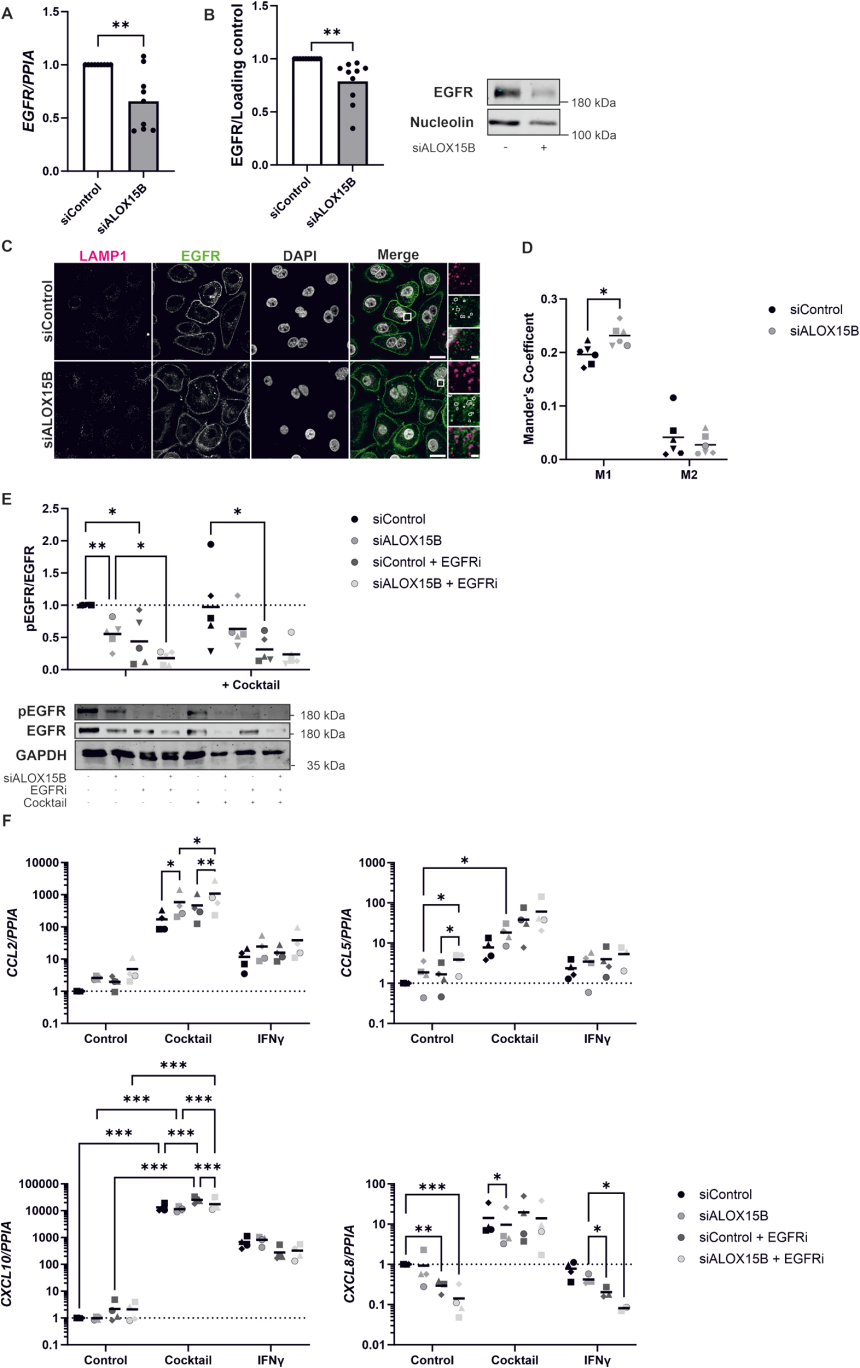
ALOX15B silencing reduces EGFR expression. Keratinocytes transfected with ALOX15B siRNA for 72 h alone (**A**–**D**) or followed by 30 min pre-treatment with 2 µM PD168393 or vehicle (DMSO), then combined with cocktail treatment of 50 ng/ml IL-17A, TNFα and 10 ng/ml IFNγ for 30 min (**E**, **F**) or IFNγ alone or 6 h (**F**). Gene expression analysis is relative to control siRNA and normalised to *PPIA* (**A**: *N* = 9, **F**: *N* = 4). Western analysis of EGFR normalised to loading control (**B**, *N* = 10). Immunocytochemistry (**C**) of lysosomal marker LAMP1 (Magenta) and EGFR (Green). Scale bar 20 µm, zoomed-in region scale bar 2 µm. White boxes represent zoomed-in regions, and white overlays represent lysosomal regions. Mander’s M1 and M2 co-localisation analysis of LAMP1 and EGFR, data are mean per cell per biological replicate (*N* = 6). Western analysis (**E**) of total and phospho EGFR (Y1068) relative to control siRNA (*N* = 5). Individual biological replicates represented by different symbol sets, line is mean, dotted line represents normalisation to siControl at 1. Two-way ANOVA (**D**–**F**), one-sample t-test (A), one-sample Wilcoxon test (B); significance denoted by *P < 0.05, **P < 0.01, ***P < 0.001.

Previous studies in keratinocytes have correlated the upregulation of *CCL2, CCL5* and *CXCL10*, and the downregulation of *CXCL8* with inhibition of EGFR leading to the activation of STAT1 [17, 18]. Therefore, the expression of EGFR following ALOX15B silencing was investigated. Both gene expression (Fig. 4A) and Western analysis of EGFR revealed a significant decrease in total EGFR protein following ALOX15B silencing (Fig. 4B). Activation of EGFR causes internalisation and vesicle trafficking, provoking either its recycling back to the plasma membrane or lysosomal degradation. Therefore, dual immunocytochemistry staining of lysosomal marker LAMP1 with EGFR was performed (Fig. 4C). Co-localisation analysis revealed an increase in Mander’s M1 co-efficient (Fig. 4D) but no change in M2. The detected increase in M1 and reduction in EGFR protein indicates an increase in the trafficking of EGFR into the lysosomal region and therefore degradation of the receptor.

Next, inhibition of EGFR via PD168393 was performed in combination with ALOX15B silencing. Phosphorylation of EGFR was significantly reduced both by ALOX15B silencing and EGFR inhibition in unstimulated cells (Fig. 4E). Cocktail treatment showed no effect on EGFR phosphorylation, however, a decrease in phosphorylation was detected in cells treated with PD168393. In line with previous reports in keratinocytes [18], Western analysis of STAT1 phosphorylation was performed following 3 h of EGFR inhibition (Fig. S6) revealing an increase in STAT1 phosphorylation to a similar level found with ALOX15B silencing. Furthermore, inhibition of EGFR revealed increases in *CCL2, CCL5* and *CXCL10* to a similar level as ALOX15B silencing (Fig. 4F). Combination of ALOX15B silencing and EGFR inhibition resulted in a further increase of CCL2 and CCL5 with cocktail treatment (Fig. 4F). ALOX15B-mediated reduction of CXCL8 was attenuated by combination of ALOX15B silencing and EGFR inhibition in IFNγ treated cells (Fig. 4F).

ALOX15B silencing regulates cholesterol homoeostasis through NRF2/EGFR/ERK signalling pathway

**Fig. 5:**
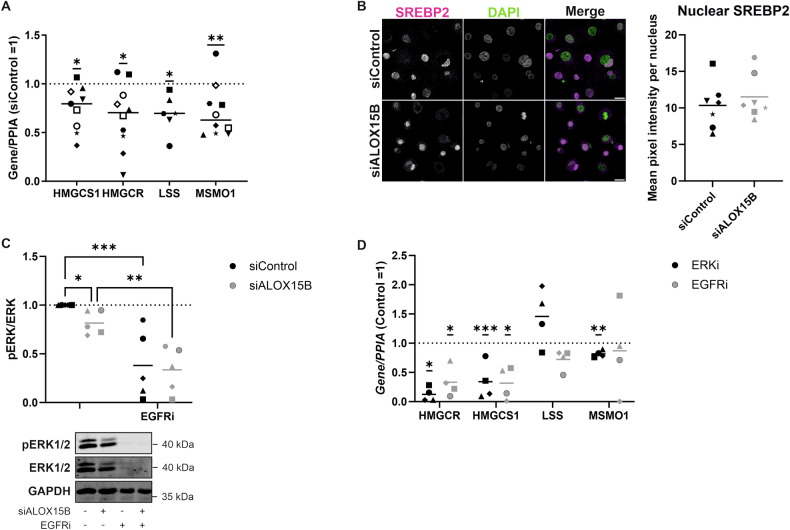
ALOX15B modulates cholesterol homoeostasis through EGFR/ERK. Gene expression analysis of cholesterol biosynthesis pathway enzymes in keratinocytes transfected with ALOX15B siRNA for 72 h (**A**) or treated with 2 µM 5 PD168393, 10 µM PD98059 or vehicle (DMSO) for 24 h. (**D**). Normalised to *PPIA* and relative to control siRNA (represented by dotted line), one sample *t* test performed (*N* = 7–9). SREBP2 (magenta) immunocytochemistry staining in keratinocytes transfected with ALOX15B siRNA for 72 h, counterstained with DAPI (green) (**B**). Scale bar 20 µm, image analysis of mean pixel intensity per nucleus. Western analysis (**C**) of total and phospho ERK (Thr202/Tyr204) relative to control siRNA (*N* = 5). Two-way ANOVA (**C**), one-sample *t* test (**A**, **D**); significance denoted by *P < 0.05, **P < 0.01, ***P < 0.001.

Recently we associated ALOX15B silencing with reduced cholesterol biosynthesis in primary human macrophages via reduced lipid peroxidation and ERK phosphorylation [19]. Therefore, expression of genes involved in cholesterol homoeostasis was examined, revealing a reduction in *HMGCS1, HMGCR, LSS* and *MSMO1* in ALOX15B silenced keratinocytes (Fig. 5A). Next, nuclear protein expression of SREBP2 was detected by confocal microscopy. Unlike in primary human macrophages [19] no reduction in nuclear SREBP2 was detected following ALOX15B silencing (Fig. 5B). Given that EGFR directly phosphorylates ERK and ERK has been associated with the transcriptional activity of SREBP2 [20], Western analysis of total and phosphorylated ERK was determined. Silencing of ALOX15B significantly reduced ERK phosphorylation. Furthermore, inhibition of EGFR reduced both total and phosphorylated ERK (Fig. 5C). To confirm the link between EGFR and ERK on SREBP2, gene expression of SREBP2 target genes was analysed. Both HMGCS1 and HMGCR, the rate-limiting enzyme in the cholesterol biosynthesis pathway, were reduced following inhibition of ERK and EGFR (Fig. 5D).

**Fig. 6:**
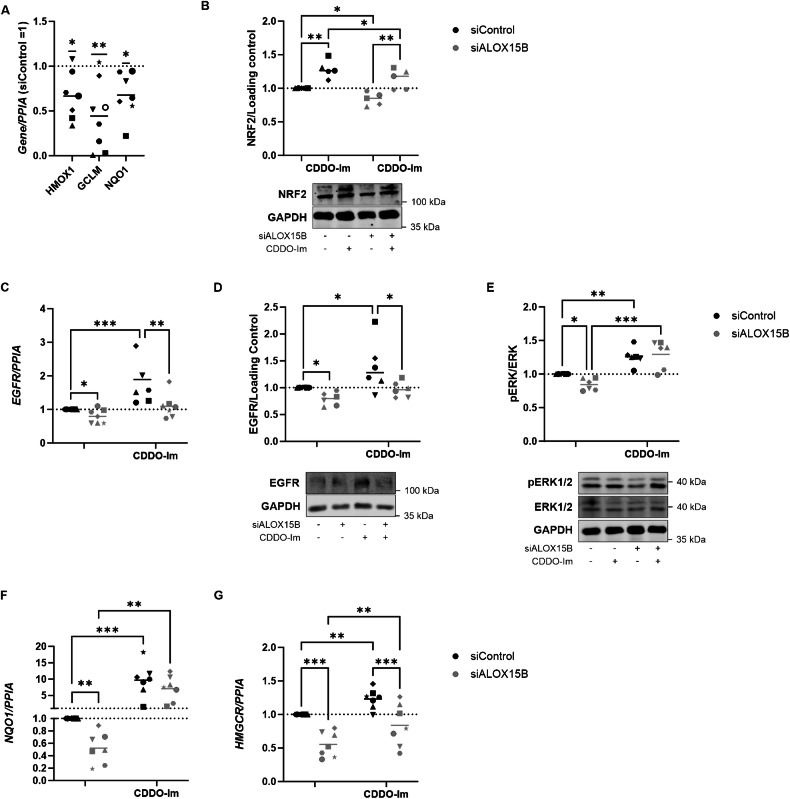
ALOX15B silencing reduces NRF2 transcription. Gene expression analysis of NRF2 target genes in keratinocytes transfected with ALOX15B siRNA for 72 h compared to siControl (**A**). Western analysis of NRF2 (**B**), EGFR (**D**) compared to total protein, phospho ERK compared to total ERK (**E**), and gene expression of EGFR (**C**), NQO1 (**F**) and HMGCR (**G**) in keratinocytes transfected with control or ALOX15B siRNA for 72 h followed by 24 h treatment with 100 nm CDDO-Imidazole (CDDO-Im). Individual biological replicates represented by different symbol sets, line is mean (*N* = 5 for gene expression and 6 for Western). *t* test (**B**, **H**) or two-way ANOVA performed (**D**–**F**, **I**); significance denoted by *P < 0.05, **P < 0.01, ***P < 0.001. For all images, scale bar 20 µm, white boxes represent zoomed-in regions.

Oxidative stress regulator NRF2 has been shown to bind to the ARE element of EGFR in melanoma cells [21]. Moreover, lipid peroxidation levels can modulate the expression of NRF2 [22, 23]. To see whether reduced lipid peroxidation detected with ALOX15B silencing also correlates with reduced NRF2 activation, gene expression of NRF2 targets was analysed. Reductions in *HMOX1, GCLM* and *NQO1* were detected following silencing of ALOX15B (Fig. 6A). Next, activation of NRF2 was performed using CDDO-imidazole (CDDO-Im) in combination with ALOX15B siRNA. A reduction in NRF2 with ALOX15B silencing was apparent via Western analysis, which was rescued with CDDO-Im (Fig. 6B). Similarly, EGFR expression was increased in cells treated with control or ALOX15B siRNA followed by CDDO-Im at both, RNA and protein level (Fig. 6C, D). In addition, phosphorylation of ERK was increased following CDDO-Im treatment, along with rescuing the ALOX15B silencing mediated reduction in ERK (Fig. 6E). To confirm that CDDO-Im treatment could activate NRF2, expression of *NQO1* was analysed. CDDO-im significantly increased *NQO1* both in control and ALOX15B transfected cells (Fig. 6F). Moreover, transcription regulation of *HMGCR* was increased from NRF2 activation (Fig. 6G).

### ALOX15B silencing alters membrane lipids

**Fig. 7:**
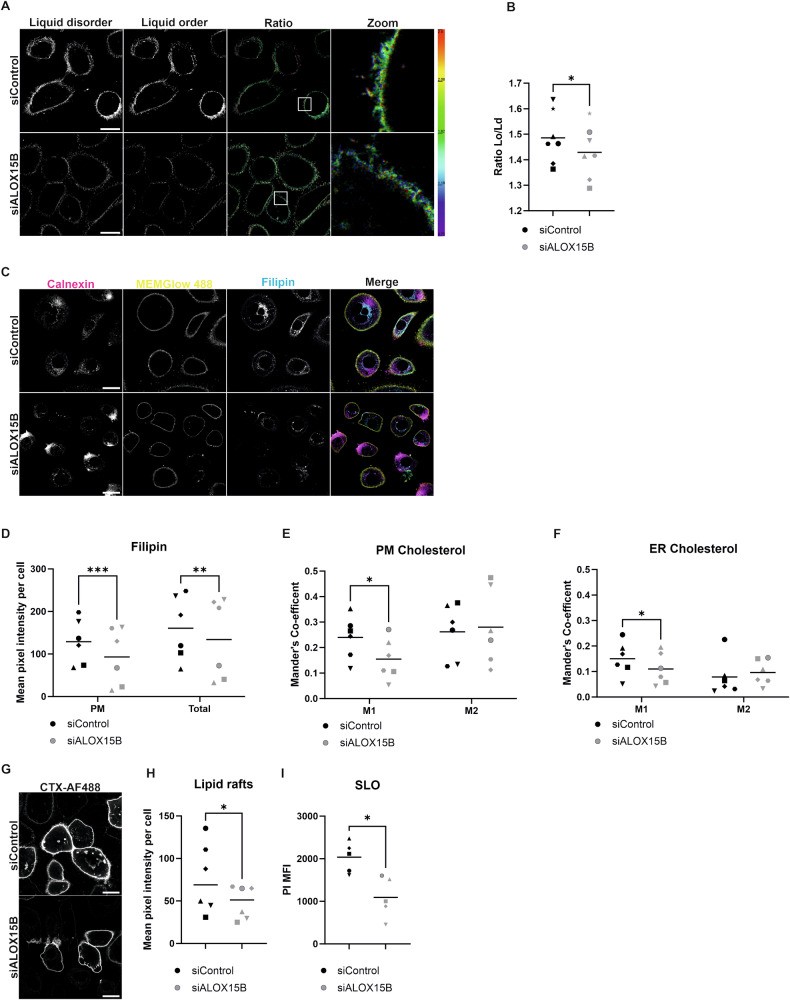
ALOX15B silencing alters membrane lipids. Analysis of membrane lipids via confocal microscopy in keratinocytes transfected with control or ALOX15B siRNA for 72 h. Ratiometric images of NR12A staining (**A**) and analysis (**B**, *N* = 7) of the ratio of liquid order to liquid disorder. Immunocytochemistry staining (**C**) of endoplasmic reticulum marker Calnexin (magenta) in combination with plasma membrane marker MemGlow 488 (yellow) and cholesterol staining by filipin (Cyan). Mean pixel intensity of filipin staining at the plasma membrane and total per cell (**D**), Mander’s M1 and M2 co-localisation analysis of MemGlow 488 (**E**) or Calnexin (**F**) and filipin, data are mean per cell per biological replicate (*N* = 6). Lipid raft staining (**G**) via Alexa Fluor 488 conjugated cholera toxin subunit B (CTX-AF488) and analysis (H) of mean pixel intensity per cell (*N* = 6). Median fluorescence intensity of propidium iodide (**I**) following 1 h streptolysin O pore formation measured by flow cytometry (*N* = 5). Individual biological replicates represented by different symbol sets, line is mean. *t* test (**B**, **H**) or two-way ANOVA performed (**D**–**F**, **I**); significance denoted by *P < 0.05, **P < 0.01, ***P < 0.001. For all images, scale bar 20 µm, white boxes represent zoomed-in regions.

Given that ALOX15B can localise to the plasma membrane in keratinocytes and has been shown to peroxidise phospholipid-bound PUFA without the need for PLA2 [24], alterations to membrane polarity were assessed. The lipid bilayer can be separated into highly regulated liquid-ordered phases (Lo) comprising of saturated phospholipids with high cholesterol and sphingomyelin concentrations or liquid-disordered (Ld) phases with unsaturated phospholipids and reduced cholesterol composition [25]. Ratiometric imaging of NR12A was performed to detect Ld at 550–600 nm and Lo at 600–650 nm (Fig. 7A). Silencing of ALOX15B revealed a significant reduction in the ratio of Lo/Ld (Fig. 7B).

As these data imply reduced cholesterol in the plasma membrane of ALOX15B silenced cells, filipin, a naturally fluorescent antibiotic produced by *Streptomyces filipinensis*, which binds to free but not esterified cholesterol was utilised. Immunofluorescence staining of the endoplasmic reticulum (the site of cholesterol biosynthesis) in conjunction with filipin and membrane marker MemGlow 488 was performed (Fig. 7C). Image analysis revealed a reduction in both total and plasma membrane intensity of filipin (Fig. 7D). Furthermore, co-localisation analysis to both plasma membrane marker (Fig. 7E) and endoplasmic reticulum marker (Fig. 7F) revealed reductions in Mander’s M1 but no change in M2, indicating reduced cholesterol but no changes to the proportion in the plasma membrane or endoplasmic reticulum.

Next, Alexa Fluor 488-conjugated cholera toxin subunit B (CTX), which binds to ganglioside GM1, was utilised as a marker of lipid rafts (Fig. 7G). Silencing of ALOX15B significantly reduced the intensity of CTX staining in keratinocytes (Fig. 7H), indicating reduced lipid raft composition in the cells. Streptolysin O binds to accessible cholesterol in the plasma membrane and forms pores, allowing the entry of propidium iodide, which normally cannot enter intact cells. Intracellular propidium iodide was detected via flow cytometry as a measure of accessible plasma membrane cholesterol. A significant reduction in propidium iodide was detected (Fig. 7I) with ALOX15B silencing, indicating reduced accessible cholesterol. Collectively, these data indicate that the silencing of ALOX15B alters plasma membrane lipids by reducing cholesterol biosynthesis.

## Discussion

**Fig. 8:**
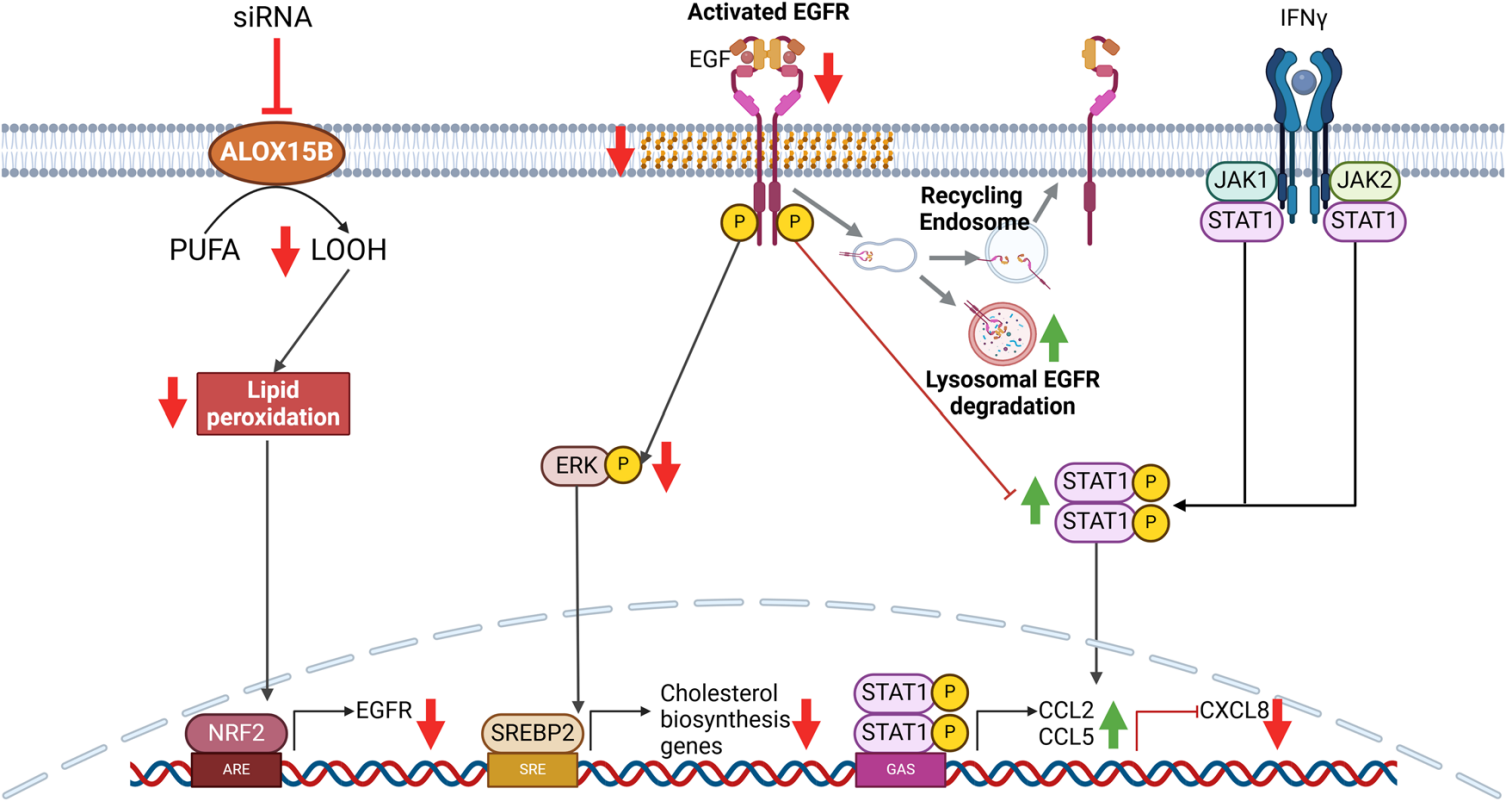
Mechanism of ALOX15B in regulation of cholesterol biosynthesis and STAT1 inflammation. Silencing of ALOX15B reduces polyunsaturated fatty acid (PUFA) derived lipid hydroperoxides (LOOH), thus reducing lipid peroxidation. Lipid peroxidation induces NRF2 activation and transcription of EGFR. ERK is phosphorylated via EGFR activation, and reduced ERK phosphorylation attenuates in the transcription of genes involved in the cholesterol biosynthesis pathway. Inhibition of EGFR activation increased STAT1 phosphorylation and in turn increased transcription and secretion of CCL2, CCL5 but reduced CXCL8. Created with BioRender.com.

Here, we provide deeper insight into the mechanism by which ALOX15B may act as an anti-inflammatory mediator in psoriasis. In line with previous reports [11], ALOX15B expression was increased in lesional psoriatic skin. Moreover, treatment with a cocktail of cytokines (IL-17A, IFNγ, TNFα) to induce a psoriasis-like phenotype in keratinocytes, increased ALOX15B RNA and protein expression. siRNA-mediated silencing of ALOX15B revealed increased cytokine secretion of CCL2, CCL5, and CXCL10. This augmentation of inflammation was associated with increased STAT1 phosphorylation and reduced EGFR expression. Recently, ALOX15B silencing in primary human macrophages was shown to reduce cholesterol biosynthesis through reduced lipid peroxidation and ERK phosphorylation [19]. The data here builds on this mechanism, showing reduction in lipid peroxidation provokes reduced ERK phosphorylation via NRF2 and EGFR signalling (Fig. 8). Collectively, this study demonstrates a role for ALOX15B in the resolution of psoriasis through modulation of EGFR and STAT1 via lipid peroxidation.

Previous studies have shown stronger expression of ALOX15B in the basal layer of the epidermis [26, 27], however, membrane localisation was shown in the differentiated layers of tissue-engineered skin [28]. Yet, *ALOX15B* expression has been observed in the basal and spinous layers of the epidermis of non-lesional skin by in situ hybridisation. In comparison, in lesional psoriatic skin, *ALOX15B* was strongly detected in the basal, spinous and granular layers [11]. In line with previous experiments in other cell types [11], Fig. S1 demonstrates that ALOX15B localises to the plasma membrane upon Ca^2+^ stimulation in keratinocytes. Given that epidermal differentiation is mediated through a Ca^2+^ gradient, membrane localisation of ALOX15B in the stratum granulosum detected in Fig. 1 aligns with this. Furthermore, expression of ALOX15B was shown to be upregulated with Ca^2+^-mediated differentiation of normal human epidermal keratinocytes [29].

The abundance of oxylipins in this serum-free cell culture model was low, nevertheless silencing of ALOX15B reduced 15-HETE and 15-HEPE. DHA derived ALOX15B product 17-HDHA was not detected, which aligns with previous reports in normal human epidermal keratinocytes from Hellmann et al. [29] where 7 days of differentiation with calcium was required for the detection of 17-HDHA. Although inhibitory activity of ML351 to ALOX15B in keratinocytes was not shown, the increased cytokine secretion found in the skin equivalents can be associated with a general decrease in lipid peroxidation. Specific ALOX15B inhibitors have been described [30, 31], but could not be used in this study as they are not commercially available. Future research should focus on knockdown/knockout of ALOX15B in more complex 3D models or in vivo.

Although activation of EGFR is accompanied by direct phosphorylation of signalling molecules, in keratinocytes, inhibitors of EGFR induce STAT1 phosphorylation [18, 32]. Inhibition of EGFR via PD168393, cetuximab or gefitinib leads to a Type I IFN response via IFNκ and therefore indirect activation of STAT1. In turn, this causes the induction of cytokines CCL2, CCL5, CXCL10 and GM-CSF, but reduced CXCL8 expression [17, 18, 32, 33]. The data presented here confirms the link between EGFR inhibition and increased STAT1 phosphorylation, but also adds an additional layer in which reduced EGFR protein and increased STAT1 phosphorylation are correlated.

The association of ALOX15B and cytokine production appears to be cell-specific. In primary human macrophages, silencing of ALOX15B reduced IL-4 stimulated cytokines (*CCL8, CCL13, CCL17, CCL18, CCL22, CCL24*) [34]. Furthermore, regulation of *CCL17* occurred SREBP2 (the master regulator of cholesterol homoeostasis)-dependently, where silencing of ALOX15B in macrophages reduces SREBP2 expression and cholesterol biosynthesis. Moreover, inhibition of 15-LOX in lung macrophages, polarised with either LPS for M1 or IL-4/IL-13 for M2 reduced CCL2, CCL3 CCL4, or CCL13, CCL18 and CCL22 production [35]. However, treatment of skin equivalents containing T cells with α-linolenic acid (ALA) reduced expression of CCL2, CXCL1, CXCL10, ICAM-1, IL-6, IL-8, TNFα, IFNγ and IL-17a [36]. Although oxylipin production was not investigated in this context, increased levels of ALA, EPA and DHA were detected following ALA supplementation, all of which are known substrates for ALOX15B [37]. Further investigation in this area using ALOX15B knockout/inhibited keratinocytes would help to determine whether the link between ω-3 PUFA supplementation and reduced cytokine production is mediated through ALOX15B or its products.

In addition to ALA, treatment of skin equivalents with ω-3 PUFAs is associated with reduced inflammation. Treatment of psoriatic skin equivalents co-cultured with T cells with EPA was shown to reduce phosphorylation of both STAT1 and STAT3 along with p65. In addition, reduced epidermal thickness and ki67+ cells were detected [38]. Whereas, DHA was shown to reduce PPARδ and activate PPARγ, in addition to reducing TNFα secretion and lowering hyperproliferation in skin equivalents [39]. EPA reduced the number of T cells in co-culture with keratinocytes derived from psoriasis patients [38]. Along with IL-17A secretion, polarisation of cells to Th1 and Th17 was reduced but an increased number of FOXP3+ (Th22) cells with EPA treatment occurred. Furthermore, a murine imiquimod-induced psoriasis model in mice expressing fat-1, an enzyme found in *Caenorhabditis elegans*, which desaturases ω-6 PUFAs into ω-3 PUFAs, resulted in reduced Th17 cells in the skin [40].

Silencing of ALOX15B in keratinocytes showed both a reduction in total and phosphorylated EGFR. Although a direct link between LOX and EGFR has not been previously made, evidence exists for the substrates or products of ALOX15B and EGFR expression or signalling. Activation of EGFR in squamous cell carcinoma keratinocytes has been shown by EPA and DHA [41], along with 15-HETE in rat vascular smooth muscle cells [42]. Exogenous 13-hydroperoxyoctadecadienoic acid or 15-HETE have been shown to enhance EGFR-mediated ERK and AKT signalling [43–47]. We have recently shown in primary human macrophages that ALOX15B silencing and inhibition with ML351 were associated with both reduced ERK and AKT phosphorylation [19]. Future experiments using exogenous oxylipins in keratinocytes would help in elucidating if a similar pattern of effects from different PUFA products also occurs in this cell type.

We recently showed in primary human macrophages that lipid peroxidation alters cholesterol homoeostasis through ERK and SREBP2 [37]. The data here confirms that both ALOX15B silencing and ERK inhibition reduces expression of SREBP2 target genes. However, SREBP2 protein localisation was not altered in keratinocytes upon ALOX15B silencing. This difference may be accounted by the differences in culture media, whereas macrophages were cultured with human serum, keratinocytes were cultured in serum-free conditions. Furthermore, ERK has been shown to directly phosphorylate SREBP2 [48] along with reducing sumoylation leading to increased transcriptional activation [49]. These changes were facilitated through insulin, however, in keratinocytes ERK phosphorylation is more sensitive to EGF than insulin stimulation [50]. Additionally, we now show the importance of ALOX15B in the activation of NRF2 and EGFR in ERK mediated regulation of SREBP2.

IL-17A is the key driver of the pro-inflammatory keratinocyte pathogenesis in psoriasis [51], along with a dominant IFNγ/STAT1 signature. Furthermore, TNFα synergises the inflammation in keratinocytes. However, IL-22 released from Th17 and Th22 cells is important in the abnormal keratinocyte differentiation found in psoriatic lesions [52]. Future work investigating ALOX15B silencing on IL-22 mediated keratinocyte inflammation should be investigated.

In conclusion, we provide evidence of an anti-inflammatory role of ALOX15B in psoriasis-like keratinocyte inflammation. Modulation of plasma membrane lipids by ALOX15B silencing led to an altered EGFR/STAT1/JAK1 signalling axis, giving insight into how PUFAs may modulate keratinocyte inflammation. The enhanced cytokine secretion from ALOX15B silencing keratinocytes implies a role in immune cell chemotaxis, which should be explored in the future.

## Materials and methods

### Materials

Human recombinant cytokines were supplied from Immunotools (Friesoythe, Germany) and reconstituted in water. JAK inhibitor I (Cayman Chemicals, Ann Arbor, Michigan, USA), EGFR inhibitor PD168393 (MedChem Express, Monmouth Junction, New Jersey, USA) and ML351 (Tocris, Wiesbaden-Nordenstadt, Germany) were dissolved in DMSO. Calcium chloride (Sigma-Aldrich, St. Louis, Missouri, USA) stock solution was prepared at 1.2 M in media. Single-use AA and linoleic acid in ethanol were supplied by Cayman Chemicals. Palmitic acid (Sigma-Aldrich) was dissolved in 0.1% fatty acid-free bovine serum albumin (Sigma-Aldrich) in media. Ascorbic acid (Sigma-Aldrich) stock solution was dissolved in media. Antibody details are available in Table S1.

### Cell culture

Primary pooled adult normal human epidermal keratinocytes (Promocell, Heidelberg, Germany) and NHEK/SVTERT3-5 (Evercyte, Vienna, Austria) were cultured in keratinocyte growth media 2 (Promocell) containing supplementMix (Promocell) and 50 µg/ml G418 (InvivoGen, Toulouse, France) termed KGM2. Subculture was achieved through incubation with 1x TrypLE Express (Gibco™, ThermoFisher, Waltham, Massachusetts, USA,) and cells were seeded at 2×10^4^ cells per cm^2^ prior to transfection. HDF/TERT164 (Evercyte) were cultured in Advanced DMEM/F12 (Gibco™) containing 10% foetal bovine serum (Capricorn Scientific, Ebsdorfergrund, Germany), 100 µg/ml G418 (InvivoGen, Toulouse, France) and 100 µg/ml ascorbic acid (Sigma-Aldrich) termed HDF media. Cell lines were purchased in April 2022 and routine mycoplasma testing was performed using Vendor®GeM clasic (Minerva Biolabs, Berlin, Germany).

### siRNA transfections

Non-targeting (D-001810-10) and ALOX15B (L-009026-00-0005) SMARTpool ON-TARGETplus™ siRNA (Horizon, Cambridge, UK) were transfected at 10 nM using Lipofectamine™ RNAiMAX Transfection Reagent (Invitrogen™) in Opti-MEM™ (Gibco™) for 8 h before replacing with fresh KGM2. For double siRNA transfections, 20 nM of non-targeting siRNA was used for control, with an additional 10 nM of non-targeting siRNA added to each of ALOX15B and STAT1 (M-003543-01) single transfections.

### Human skin equivalents

HDF/TERT164 cells were seeded at 1×10^7^ cells/ml in Alvatex scaffolds (Repocell, Maryland, USA) for 90 min. Wells were then flooded with HDF media containing 5 ng/ml TGFβ1. Media was changed every 3 days for 28 days. NHEK cells were seeded at 1.5×10^7^ cells/ml onto the scaffolds with KGM2 media in the top compartment and HDF media with TGFβ1 in the outer compartment for 3 days. Subsequently, skin equivalents were lifted to an air-liquid interphase by culturing with only KGM2 media containing 100 µg/ml ascorbic acid, 1.64 mM CaCl_2_ and a lipid mix (30 µM linoleic acid, 25 µM palmitic acid, 10 µM AA in 0.1% fatty acid-free bovine serum albumin) and cultured for 14 days. Then treated with cytokine cocktail with or without 10 µM ML351 (Cayman chemicals) for 2 days.

### RNA isolation and qPCR

Cells seeded into 24 well plates were lysed with 300 µl TRIzol™ (Invitrogen™) and RNA was isolated as per manufacturer recommendations. Maxima First Strand cDNA Synthesis Kit (ThermoFisher Scientific) was used to convert RNA into cDNA. 10 ng cDNA was used for qPCR using Power up SYBR green master mix (Applied Biosystems™). Analysis was performed using the ΔΔCT method using *PPIA* as a reference gene. Primer (Biomers.net, Ulm, Germany) sequences are available in Table S2.

### Flow cytometry

For cytokine analysis media from cell cultures or skin equivalents was used with BD™ Cytometric Bead Array (CBA) Human Flex Sets (BD Biosciences, Franklin Lakes, New Jersey, USA) as per manufacturers’ recommendations. For membrane cholesterol measurements, streptolysin O (Sigma-Aldrich) assay was performed as previously described [53]. Samples were measured using FACS Symphony A5 (BD biosciences). Analysis was performed using FlowJo (BD Biosciences).

### Western blotting

Cells were lysed in urea buffer containing protease and phosphatase inhibitors and sonicated. 50 µg of protein were run on 10% tris-glycine gels and transferred onto nitrocellulose membranes using a Trans-Blot Turbo transfer system (Bio-Rad, Hercules, California, USA). Membranes were stained with Revert™ 700 Total Protein Stain (LI-COR, Lincoln. Nebraska, USA) as per manufacturer recommendations. Membranes were cut to detect different proteins. Blocking was performed with 5% bovine serum albumin in TBST for 1 h before incubations with primary antibodies at 4 °C overnight. Membranes were incubated with either IRDye® 800CW Goat anti-Rabbit IgG Secondary Antibody or IRDye® 680RD Goat anti-Mouse IgG Secondary Antibody (LI-COR) for 1 h at room temperature. Membranes were scanned with Odyssey CLx (Licor). Densitometry analysis was performed in ImageStudio ™ Lite (LI-COR). When more than one rabbit primary antibody was used, membranes were stripped with 25 mM Glycine + 2% SDS pH 2 buffer for 15 min before blocking and re-probing. Secondary antibody IRDye® 680RD Goat anti-Rabbit IgG Secondary Antibody (LI-COR) was subsequently used.

### Immunocytochemistry and fluorescence staining

Cells were seeded onto poly-l-lysine coated µ-Slide 8 Well Glass Bottom (ibidi, Gräfelfing, Germany) and transfected as described above. For lipid peroxidation experiments cells were incubated with 10 μM BODIPY 581/591 C11 (ThermoFisher Scientific) in media for 30 min at 37 °C, in addition 10 μM ML351 was added for the inhibitor experiment. Subsequently cells were washed twice with PBS and imaged immediately. For lipid order measurements, cells were stained with 20 nM MemGlow™ NR12A Membrane Polarity Probe (Cytoskeleton, Denver, Colorado, USA) for 10 min at room temperature. For lipid rafts, cells were stained with 2.5 µg/ml cholera toxin for 10 min on ice and then fixed with 1% paraformaldehyde. For immunocytochemistry cells were fixed in 4% paraformaldehyde and permeabilised with 0.1% triton X (except for filipin staining), blocked with 10% normal goat serum containing 100 mM Glycine and incubated with primary antibodies (with 100 μg/ml filipin (Sigma-Aldrich) for filipin staining) at 4 °C overnight. The following day cells were incubated with 1:500 secondary antibody for 45 min and counterstained with 20 nM MEMGlow 488 (Cytoskeleton) for 10 min and 1 µg/ml DAPI for 1 min.

### Confocal microscopy

Images were acquired using Zeiss LSM 800 Axio-Observer with Plan-Apochromat 40×/1.4 Oil DIC M27 objective and GaAsP detector. Acquisition and airyscan processing was performed using Zen Blue software. Detailed settings are available in Table S3.

### Patient sample collection and ethics

3 healthy volunteers and 3 psoriasis patients gave written informed consent for skin biopsies. The study was approved by the ethics committee of the Clinic of the Goethe-University (116/11); written, informed consent was obtained from all patients and control participants. The Declaration of Helsinki protocols were followed. Punch biopsies (6 mm) were taken, fixed in 4% PFA and paraffin embedded. 4 µm sections were processed routinely.

### RNA scope and fluorescence immunohistochemistry

Tissue sections were probed with RNAscope™ Probe- Hs-ALOX15B (420171, ACDBio) using RNAscope™ Multiplex Fluorescent Assay kit as per manufacturer recommendations. The ALOX15B probe was detected using Opal 620 (Akoya biosciences, Marlborough, Massachusetts, USA) diluted at 1:300. Subsequently, slides were microwaved in AR9 buffer (Akoya biosciences) for 15 min at 250 W, blocked with BLOXALL® (Vector Laboratories, California, United States) and incubated with primary antibodies overnight at 4 °C. Slides were incubated with polyclonal Goat anti-Rabbit Immunoglobulins/HRP (1:500; P0448, DAKO, Agilent, Santa Clara, California, USA) for 10 min. Rabbit HRP was detected using 1:300 Opal 570 in TSA dilatant (Akoya Biosciences) for 10 min. The remaining HRPs were blocked with HRP blocker (RNAscope kit, ACDbio) for 10 min. Slides were then incubated with polyclonal Goat anti-Mouse Immunoglobulins/HRP (1:500; P0447, DAKO) for 10 min. Mouse HRP was detected using 1:300 Opal 690 in TSA dilatant (Akoya biosciences) for 10 min. Slides were counterstained with DAPI (RNAscope kit, ACDbio) for 1 min. Slides were imaged using VectraPolaris at x40 magnification (Akoya Biosciences). Heamatoxylin and Eosin staining was performed using H&E fast staining kit (Carl Roth, Karlsruhe, Germany) as per manufactures recommendations.

### Image analysis

Macros were written for intensity analysis per cell or nucleus in ImageJ FIJI [54]. BIOP JaCoP plugin was used for Mander’s co-localisation analysis. For NR12A images, the ratio was made through dividing the intensities per cell of Ld at 550-600 nm and Lo at 600-650 nm and ratiometric images were generated using the RatioloJ plugin [55]. Qupath [56] was used to generate ROIs for the epidermis to measure intensity and subcellular detection for the number of RNAscope spots. Epidermis thickness was quantified in QuPath using the length measurement from 12 line profiles per skin equivalent. Image analysis scripts are made available upon request.

### Oxylipin analysis

Following cell treatments, keratinocytes were washed once with PBS and scraped in ice-cold PBS. Cells were centrifuged at 2400 g at 4 °C for 5 min, and pellets were snap-frozen with liquid nitrogen. Cell pellets were resuspended in methanol/H_2_O (50/50 v/v), antioxidant and inhibitor solution was added and suspension was sonicated for 8 s using an ultrasonic tip as described [57, 58]. Protein content was determined via BCA assay [59] using BCA reagent A from Fisher Scientific (Schwerte, Germany). After the addition of internal standards, proteins were precipitated with ice-cold isopropanol for 30 min at -80 °C. Following centrifugation at 20 000 g for 10 min at 4 °C supernatants were saponified with 0.6 M potassium hydroxide in methanol/H_2_O (75/25 v/v) for 30 min at 60 °C [60]. After neutralisation and purification using solid phase extraction, oxylipins were quantified by LC-MS/MS as described [57, 61].

### Statistics

All experiments performed had a minimum of 3 biological replicates from individual passages of cells, sample size was not pre-determined. Statistical analysis and data visualisation were performed using Prism (GraphPad, Boston, Massachusetts, USA). Data are presented as scatter plots with mean, biological replicates represented by different symbol sets. Normality testing was performed using Shapiro-Wilk testing, for data which did not follow a Gaussian distribution, non-parametric equivalents were performed. One-sample *t* tests, paired one-tailed *t* tests, Wilcoxon test or two-way ANOVA were performed as stated in the figure legends. Statistical significance was described as *P ≤ 0.05, **P ≤ 0.01, ***P ≤ 0.001.

## Supplementary information

Supplementary Information

Original Western Blots

## Data Availability

Data will be made available on request.
