# Supplementary material for: RNAi-based ALOX15B silencing augments keratinocyte inflammation in vitro via EGFR/STAT1/JAK1 signalling
Source: Cell Death Dis. 2025 Jan 22;16(1):39. doi: 10.1038/s41419-025-07357-x (PMC11754432; doi:10.1038/s41419-025-07357-x)
Supplement: Supplementary file 1 — Supplementary Information [file 41419_2025_7357_MOESM1_ESM.docx]

RNAi-based ALOX15B silencing augments keratinocyte inflammation in vitro via EGFR/STAT1/JAK1 signalling

Running Title: ALOX15B knockdown augments keratinocyte inflammation

Megan A. Palmer^1^, Rebecca Kirchhoff^2^, Claudia Buerger^3^, Yvonne Benatzy^1^, Nils Helge Schebb^2^, Bernhard Brüne^1,4,5^

**
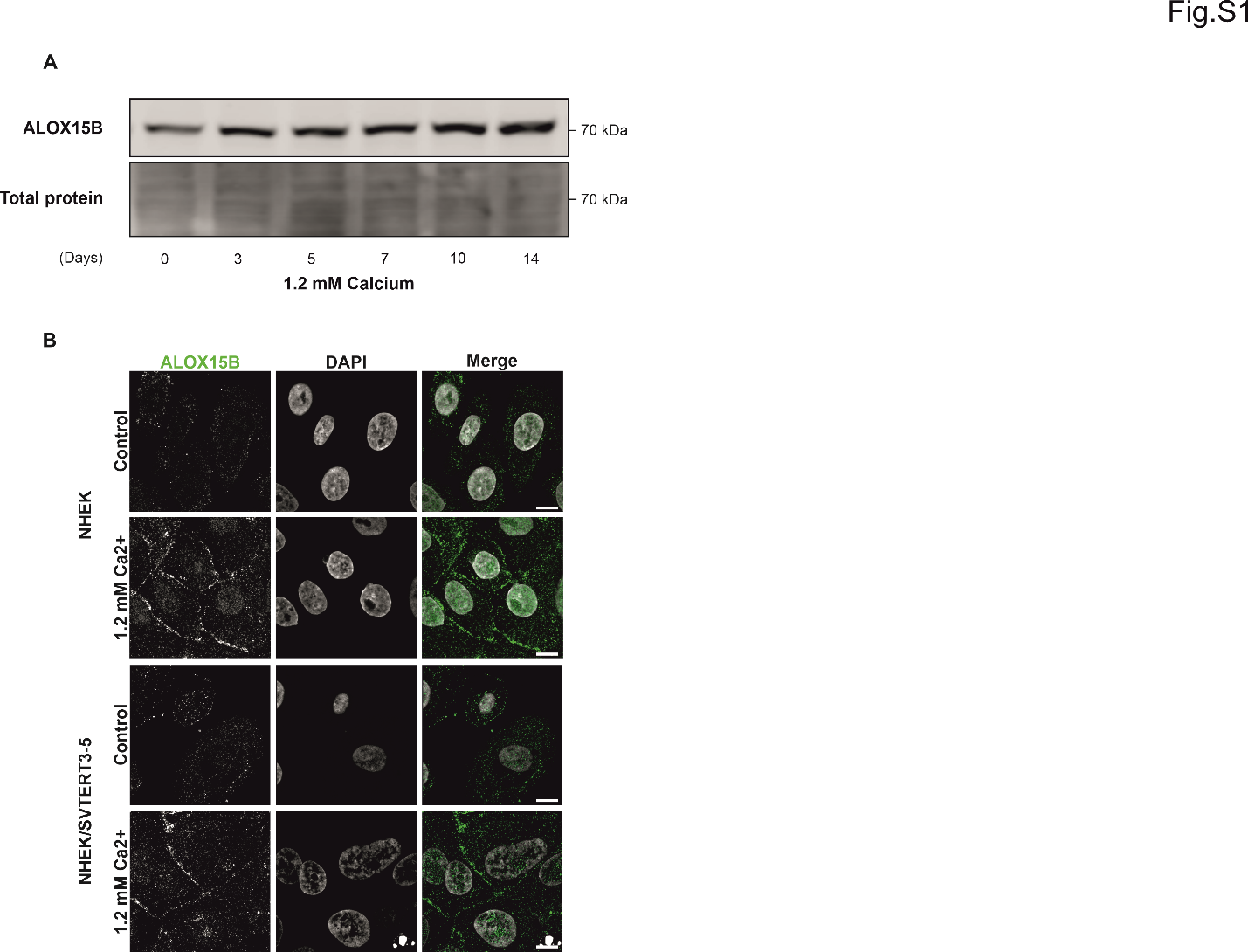
**

**Fig. S1 ALOX15B localises to the plasma membrane following calcium treatment.** Protein analysis of ALOX15B detected by Western analysis (A) or immunocytochemistry (B) in primary normal human epidermal keratinocytes or immortalised keratinocyte cell line NHEKSV/TERT3-5treated with 1.2 mM Ca^2+^ for 0-14 days or 5 days (B). Scale bar 10 µm.

**
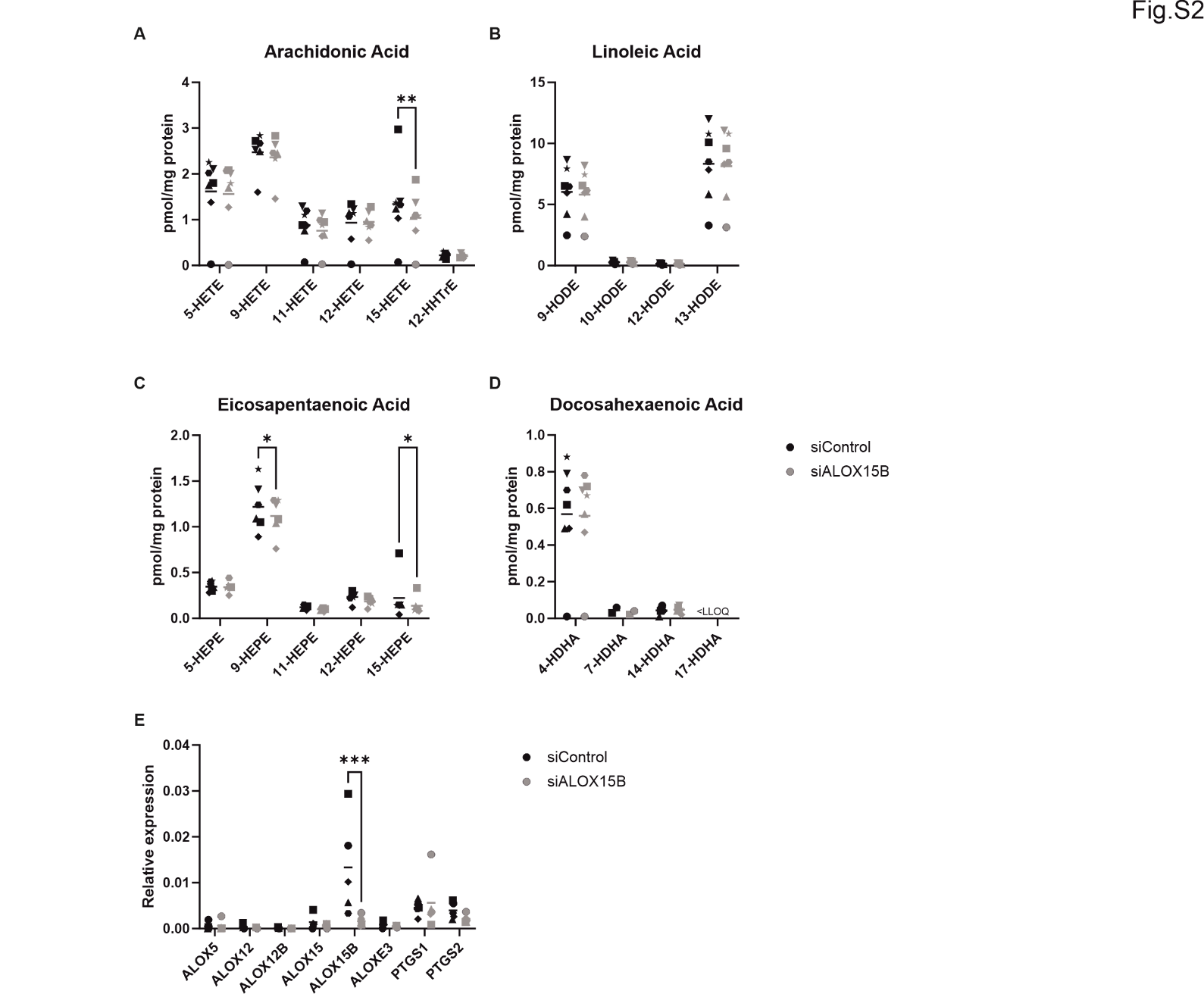
**

**Fig. S2 Oxylipin profile in keratinocytes.** Analysis of oxylipins (A-D) and gene expression of lipoxygenases and cyclooxygenases (E) in keratinocytes transfected with ALOX15B siRNA for 72h. Double hydroxylated oxylipins from (i.e. 5,12- and 5,15-diHETE), EPA (i.e. 5,15-diHEPE) and DHA (i.e. 7,17- and 10,17-diHDHA) were below the lower limit of quantification (<LLOQ). Gene expression is relative to *PPIA.* Individual biological replicates represented by different symbol sets, line is mean, N=7 for oxylipin analysis and N=5 for gene expression. Two-way ANOVA performed, significance denoted by *** P < 0.001.


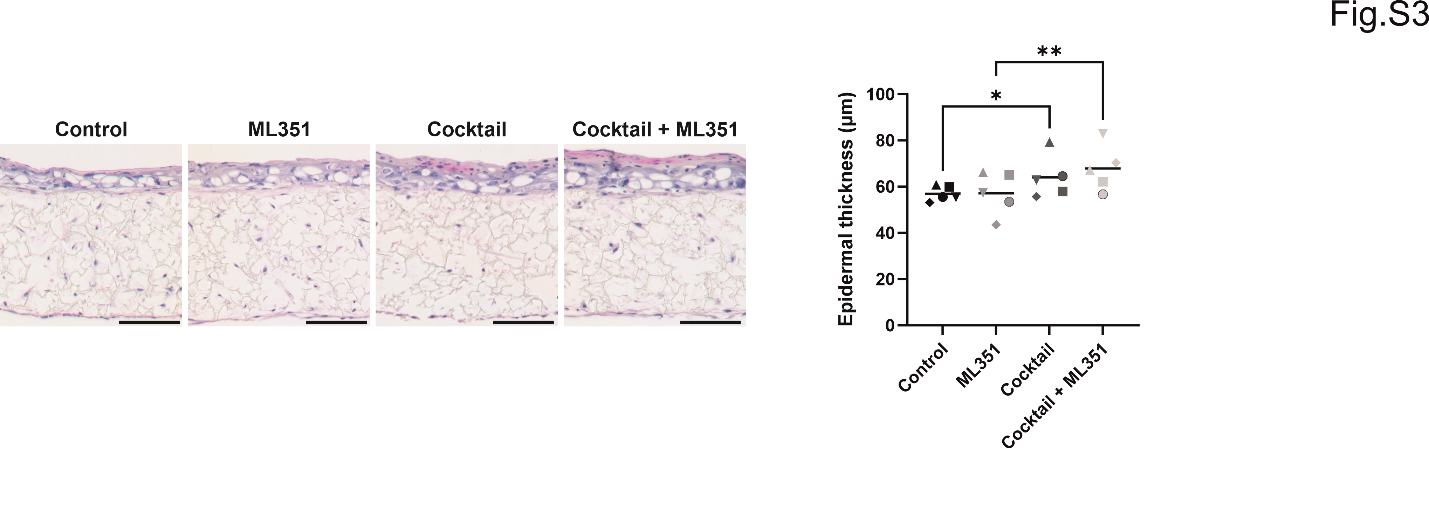
**Fig. S3 Epidermal thickening in human skin equivalents treated with cytokine cocktail.** Haematoxylin and eosin staining in human skin equivalents treated with 10 µM ML351 or vehicle (DMSO) for 48h with or without cocktail treatment of 50 ng/ml IL-17A, TNFα and 10 ng/ml IFNγ. Epidermal thickness was measured at 12 locations throughout the epidermis and averaged (N = 5). Individual biological replicates represented by different symbol sets, line is mean. Two-way ANOVA performed, significance denoted by * P < 0.05, ** P < 0.01. Scale bar 100 µm.


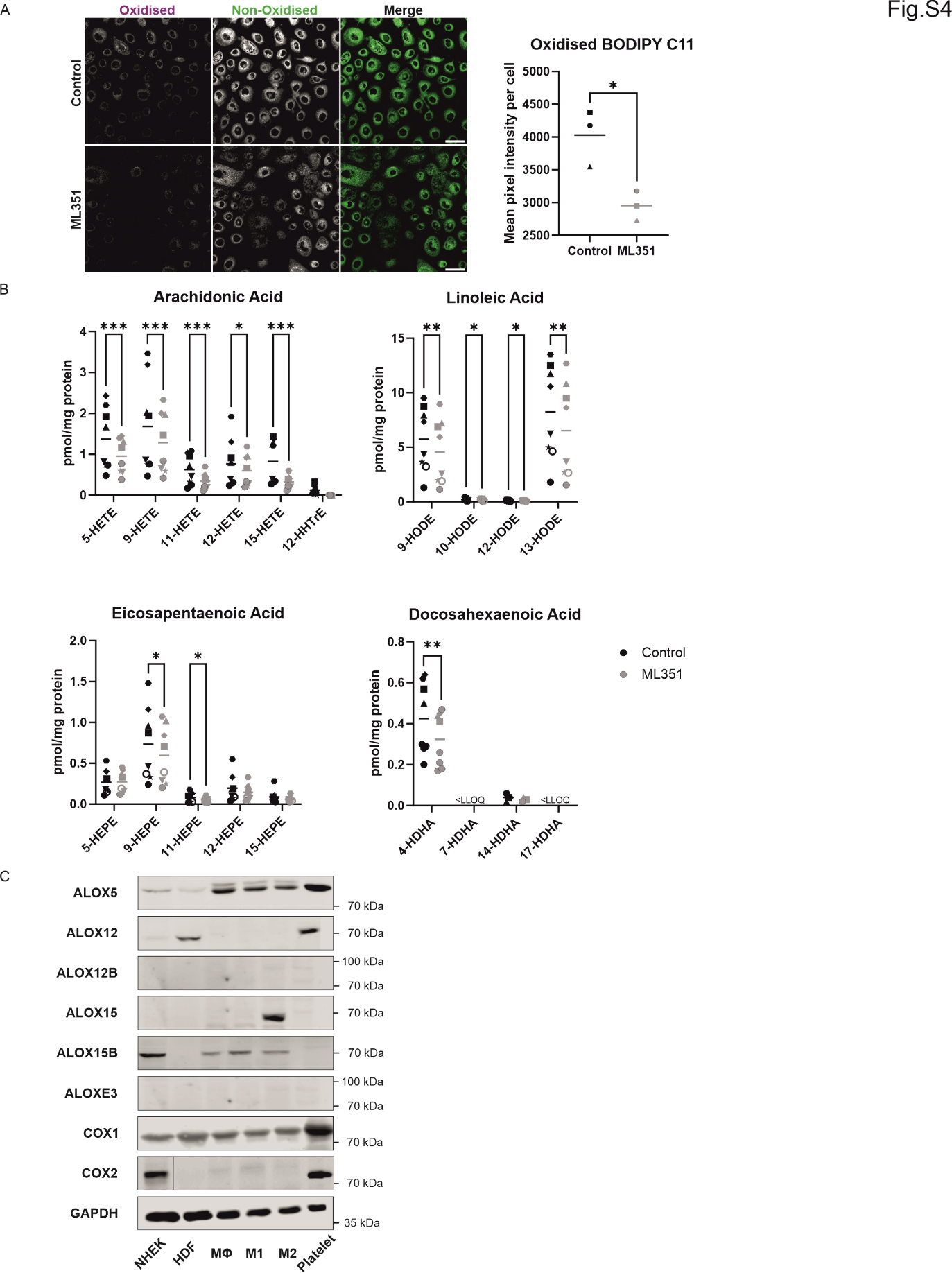


**Fig. S4 ML351 reduces lipid peroxidation in keratinocytes.** (A) Confocal microscopy of lipid peroxidation sensor BODIPY C11 in keratinocytes treated with 10 µM ML351 or vehicle (DMSO) 24h (B). Scale bar 50 µm, image analysis of mean pixel intensity per cell of oxidised BODIPY C11 (N=3). Concentrations of oxylipins (B) in keratinocytes treated with 10 µM ML351 or vehicle (DMSO) 24h (N=8). Double hydroxylated oxylipins from (i.e. 5,12- and 5,15-diHETE), EPA (i.e. 5,15-diHEPE) and DHA (i.e. 7,17- and 10,17-diHDHA) were below the lower limit of quantification (<LLOQ). Two-way ANOVA performed for oxylipins and t-test for oxidised BODIPY C11 analysis, significance denoted by * P < 0.05, ** P < 0.01, *** P< 0.001. Western analysis (C) of lipoxygenase and cyclooxygenases in keratinocytes, fibroblasts, macrophages and platelets.


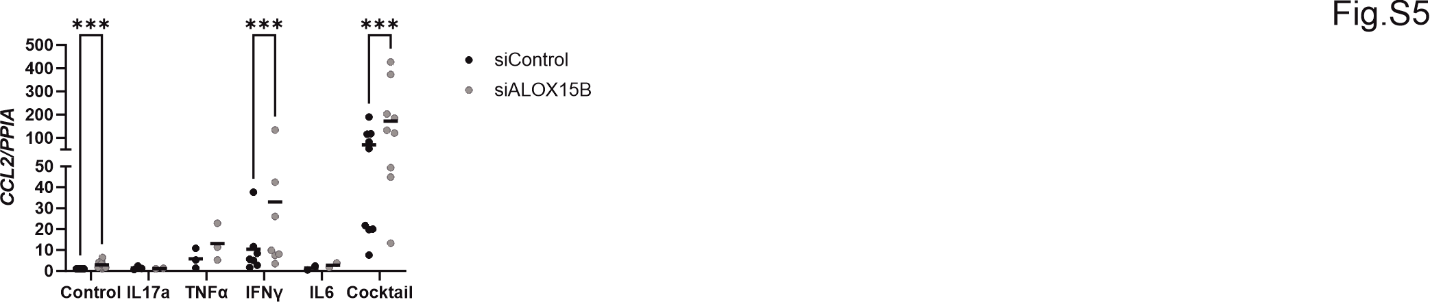


**Fig. S5 IFNγ mediates ALOX15B induced CCL2 expression.** Gene expression analysis of *CCL2* in keratinocytes transfected with control or ALOX15B siRNA for 72h, followed by a cocktail of 50 ng/ml IL-17A, TNFα and 10 ng/ml IFNγ or 50 ng/ml IL-6 for 6h. Data normalised to *PPIA* and relative to control siRNA (N=2-9). Data representative with line as mean, two-way ANOVA was performed; significance denoted by *** P < 0.001.


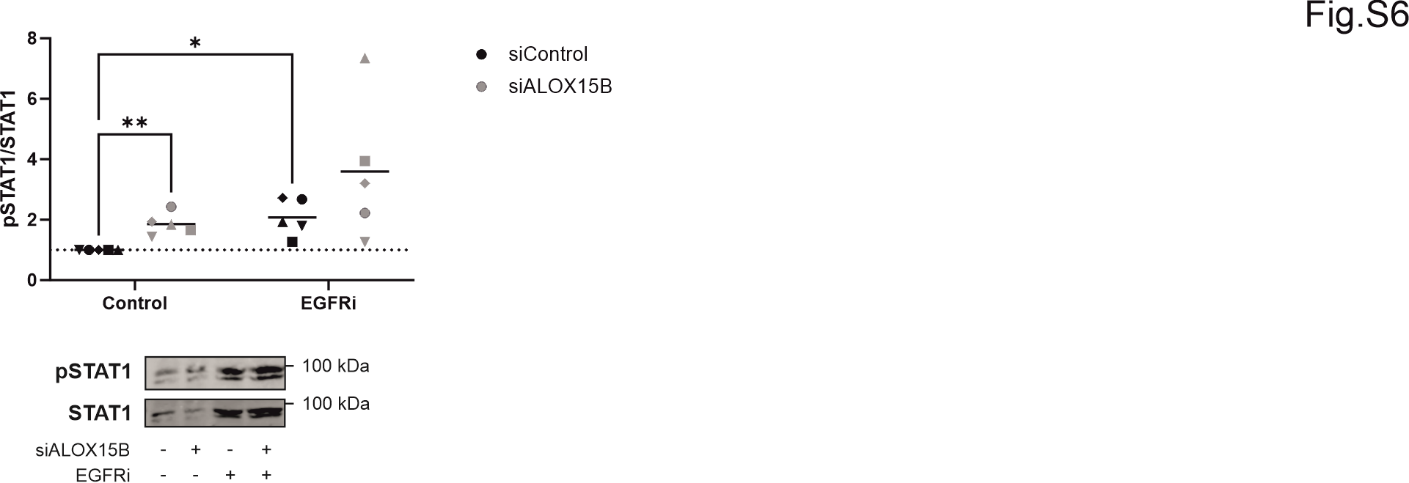


**Fig. S6 STAT1 phosphorylation is increased with EGFR inhibition.** Western analysis of total and phospho STAT1 in keratinocytes with knockdown of ALOX15B for 72h followed by 3h treatment with 2 µM PD1578780 or vehicle (DMSO). Western analysis is normalised to siControl cells (represented by dotted line at 1) (N=5). Individual biological replicates represented by different symbol sets. Two-way ANOVA performed; significance denoted by * P < 0.05, ** P < 0.01, *** P < 0.005.

**Table. S1: Antibodies**

| **Antibody** | **Clone** | **Application** | **Species** | **Dilution** | **Product code** |
| --- | --- | --- | --- | --- | --- |
| ALOX5 | 33/5-Lipoxygenase | Western | Mouse | 1:1000 | 610694 (BD Biosciences) |
| ALOX12 | Polyclonal | Western | Rabbit | 1:1000 | ab211506 (abcam) |
| ALOX12B |  | Western | Rabbit | 1:1000 | HPA024002 (Sigma) |
| ALOX15 | EPR22138 | Western | Rabbit | 1:1000 | ab244205 (abcam) |
| ALOX15B | Polyclonal | Western | Rabbit | 1:1000 | 10004454 (Cayman Chemicals) |
| ALOX15B | Polyclonal | IHC | Rabbit | 1:2000 | PAB13608 (Abnova) |
| ALOXE3 | Polyclonal | Western | Rabbit | 1:1000 | ab118470 (abcam |
| Calnexin | 1C2.2D11 | ICC | Rabbit | 1:100 | NBP2-36570 (Novus Biologicals) |
| CD68 | PG-M1 | ICC | Mouse | 1:100 | M087601-2 (Dako) |
| COX1 | EPR5866 | Western | Rabbit |  | ab109025 (abcam) |
| COX2 | D5H5 | Western | Rabbit | 1:1000 | 12282 (Cell signalling) |
| EGF Receptor | D38B1 | Western | Rabbit | 1:1000 | 4267 (Cell signaling) |
| EGF Receptor | D38B1 | ICC | Rabbit | 1:200 | 4267 (Cell signlling) |
| p-p44/42 MAPK (Erk1/2) (Thr202/Tyr204) | Polyclonal | Western | Rabbit | 1:1000 | 9101(Cell signlling) |
| p44/42 MAPK (Erk1/2) | 137F5 | Western | Rabbit | 1:1000 | 4695 (Cell signlling) |
| p-EGF Receptor  (Tyr1068) | D7A5 | Western | Rabbit | 1:1000 | 3777 (Cell signaling) |
| GAPDH | 1E6D9 | Western | Mouse | 1:50000 | 60004-1-lg (Proteintech) |
| LAMP1 | H4A3 | ICC | Mouse | 1:100 | ab25630 (abcam) |
| NRF2 | Polyclonal | Western | Rabbit | 1:6000 | 16396-1-AP (Proteintech) |
| C23 (Nucleolin) | H-6 | Western | Mouse | 1:1000 | sc-5586 (Santa Cruz) |
| SREBP2 | Polyclonal | ICC | Rabbit | 1:500 | 10007663 (Cayman chemicals) |
| STAT1 | 9H2 | Western | Mouse | 1:1000 | 9176 (Cell signaling) |
| p-STAT1 | D4A7 | Western | Rabbit | 1:1000 | 7649 (Cell signalling) |
| F(ab')2-Goat anti-Mouse IgG Alexa Fluor™ 647 |  | ICC |  | 1:500 | A21237 (Invitrogen) |
| F(ab')2-Goat anti-Rabbit IgG Alexa Fluor™ Plus 555 |  | ICC |  | 1:500 | A48283 (Invitrogen) |

ICC: immunocytochemistry, IHC: Immunohistochemistry

**Table. S2: Primer sequences**

| **Primer** | **Forward** | **Reverse** |
| --- | --- | --- |
| *ALOX15B* | 5'-aag ggc ttc cta aac cag ga-3' | 5'-tga cat cac atg tgg cat tg-3' |
| *CCL2* | 5'-tga tcc caa tga gtc ggc tg-3' | 5'-tgg acc cat tcc tta ttg ggg-3' |
| *CCL5* | 5'- ctg cat ccc tca ccg tca tc-3' | Rev: 5'- gcg gtt cct tcg agt gac aa-3' |
| *CXCL10* | 5'-gtg gca ttc aag gag tac ctc -3' | 5'-tga tgg cct tcg att ctg gat t |
| *CXCL8* | 5'-ctg cgc caa cac aga aat ta-3' | 5'-att gca tct ggc aac cct ac-3' |
| *EGFR* | 5'-ttg ccg caa agt gtg taa cg-3' | 5'- gtc acc cct aaa tgc cac cg-3' |
| *GCLM* | 5’-cct gtt cag tcc ttg gag ttg c-3’ | 5’- ctg tgt ttt gtc tag atc aga ggt acc tat g-3’ |
| *HMGCS1* | 5'-cat tag acc gct gct att ctg tc-3', | 5'-ttc agc aac atc cga gct aga-3' |
| *HMGCR* | 5'-tga ttg acc ttt cca gag caa g-3' | 5'-cta aaa ttg cca ttc cac gag c-3' |
| *HMOX1* | 5’-act gcg ttc ctg ctc aac at-3’ | 5’-ggg cag aat ctt gca cttt gtt-3’ |
| *IL6* | 5'-tcc aca agc gcc ttc ggt cc-3' | 5'-tcc aca agc gcc ttc ggt cc -3' |
| *LSS* | 5'-gca ctg gac ggg tga tta tgg-3' | 5'- tct ctt ctc tgt atc cgg ctg-3' |
| *MSMO1* | 5'-tat gct ggt tct cgg cat cat-3' | 5'-cca aaa att cga tcc cac cat gt-3' |
| *NQO1* | 5’-tga aag gct ggt ttg agc ga-3’ | 5’-gcc ttc tta ctc cgg aag gg-3’ |
| *PPIA* | 5’-cgc cga gga aaa ccg tgt a-3’ | 5’-acct tgt ctg caa aca gct ca-3’ |

**Table. S3: Confocal microscopy settings**

| **Staining** | **Laser** | **Detector gain** | **Emission** | **Pinhole** | **Scaling** |
| --- | --- | --- | --- | --- | --- |
| BODIPY C11 | 488 nm (0.2%)  561 nm (0.4%) | 800 V  650 V | 400-552  579-700 | 33 µm | 0.085 x 0.085 µm |
| SREBP2  DAPI | 561 nm (2.9%)  405 nm (1.4%) | 800 V  800 V | 535-617  400-600 | 31 µm  27 µm | 0.071 x 0.071 µm |
| NR12A | 488 nm (0.8%) | 850 V | 600-650  550-600 | 191 µm  175 µm | 0.061 x 0.061 µm |
| CTX-AF488 | 488 nm (0.85%) | 760 V | 400-700 | 158 µm | 0.043 x 0.043 µm |
| LAMP1  EGFR  DAPI | 640 nm (0.9%)  561 nm (0.3%)  405 nm (0.4%) | 900 V  800 V  900 V | 617-700  540-620  400-600 | 178 µm  157 µm  154 µm | 0.035 x 0.035 µm |
| Filipin  Calnexin  MEMGlow 488 | 640 nm (0.5%)  488 nm (0.5%)  405 nm (2%) | 850 V  850 V  900 V | 450-700  490-620  400-600 | 178 µm  147 µm  144 µm | 0.035 x 0.035 µm |
