# Supplementary figures and images for: RNAi-based ALOX15B silencing augments keratinocyte inflammation in vitro via EGFR/STAT1/JAK1 signalling
Source: Cell Death Dis. 2025 Jan 22;16(1):39. doi: 10.1038/s41419-025-07357-x (PMC11754432; doi:10.1038/s41419-025-07357-x)

Fig.1E

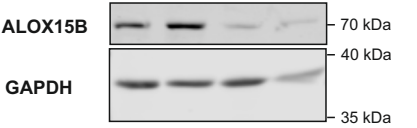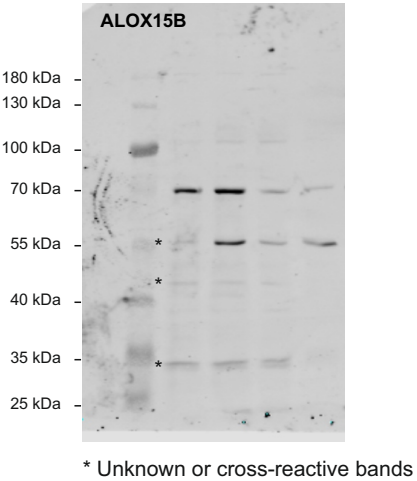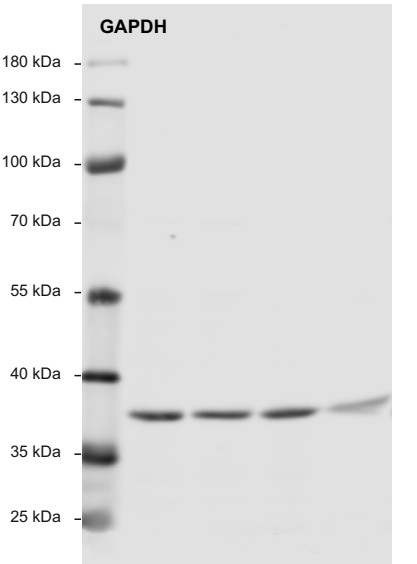

Fig.3A

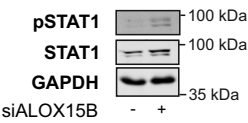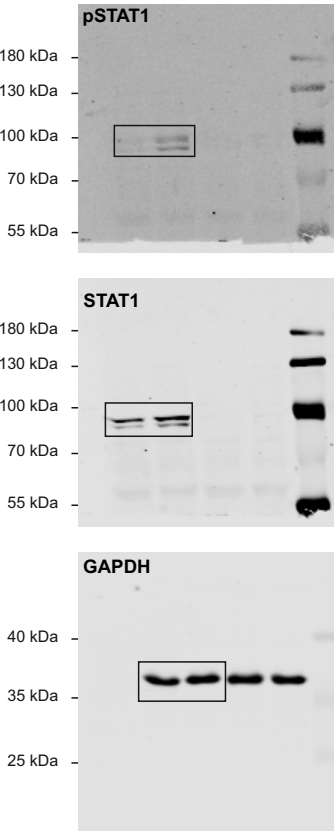

**Fig.3B**

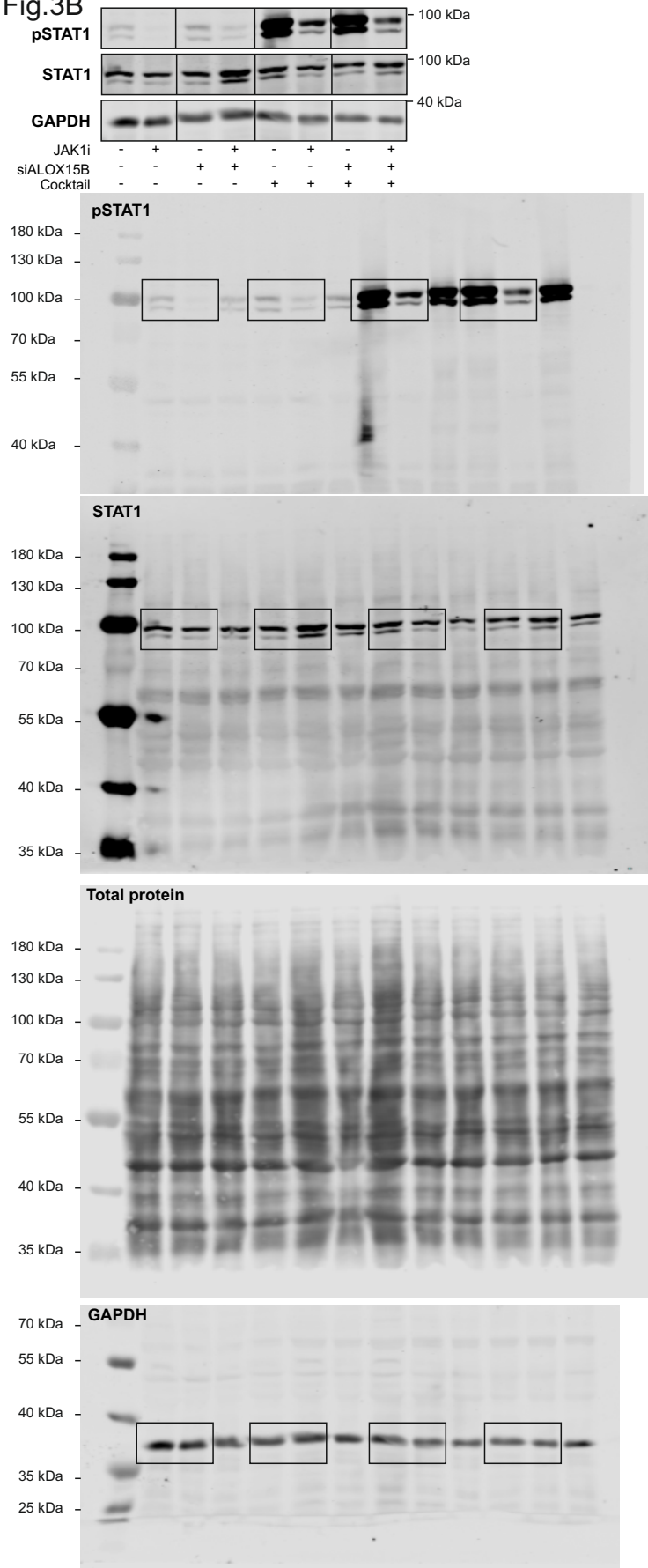

Fig.3D

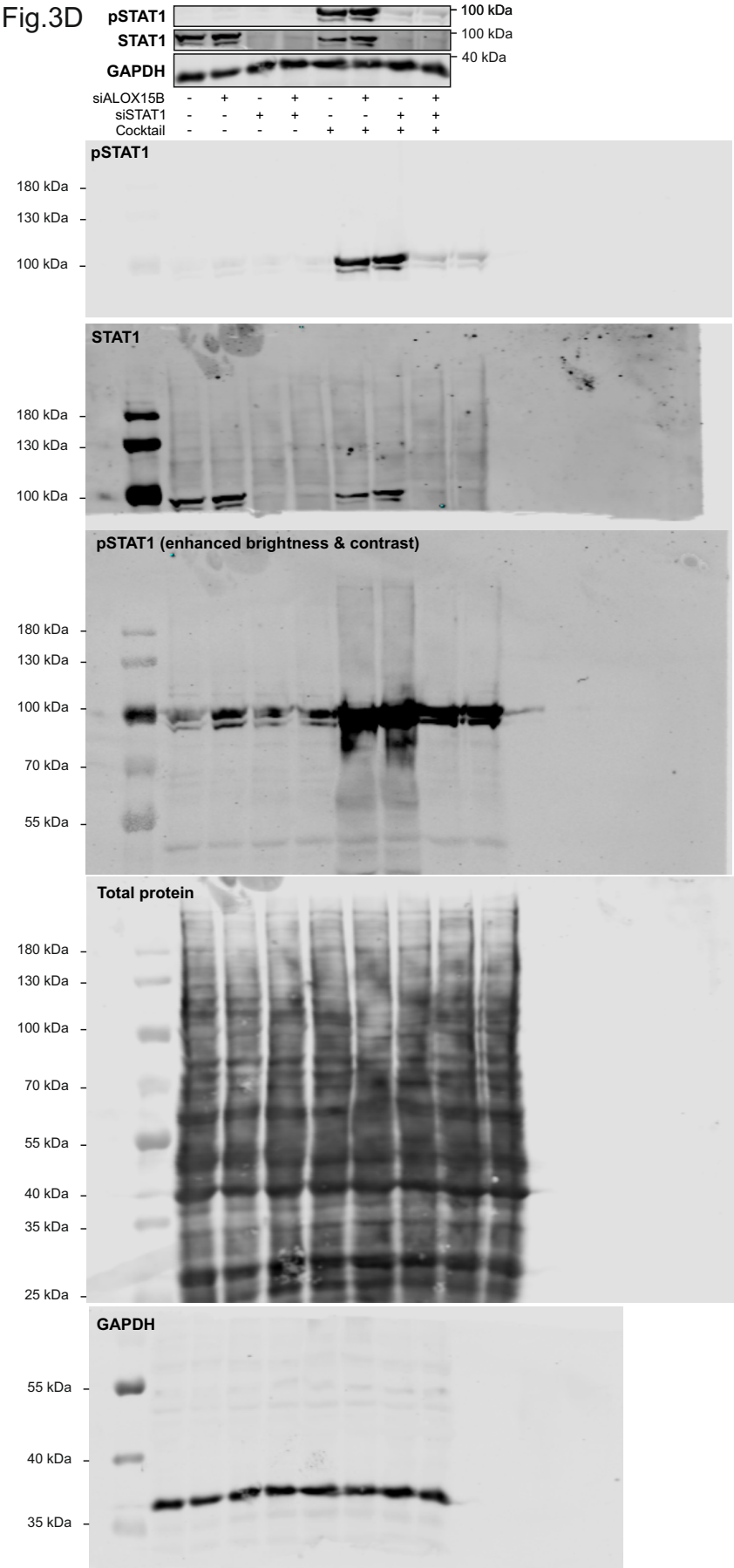

Fig.4A

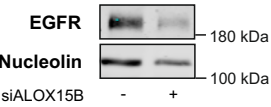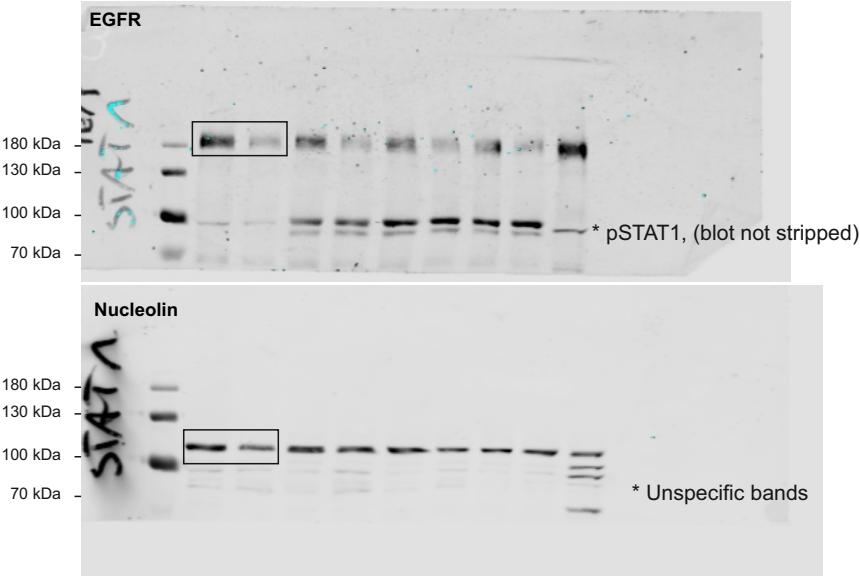

Fig.4E

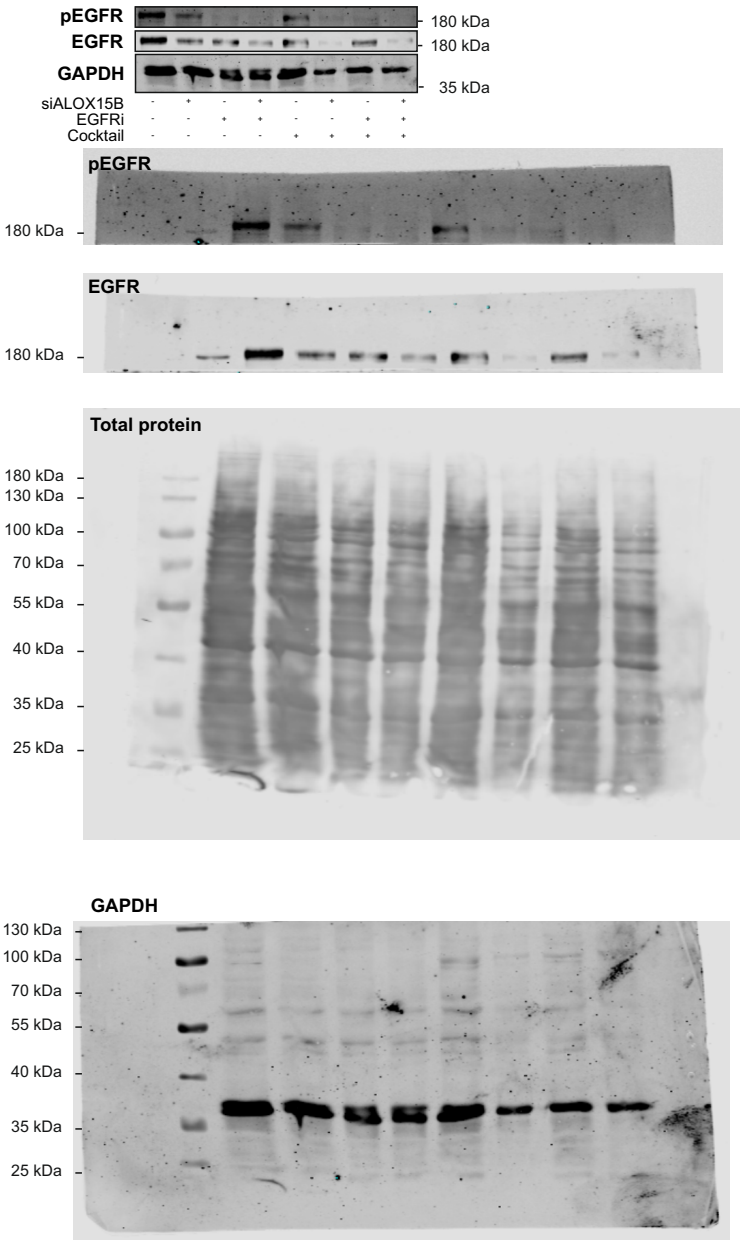

Fig.5C

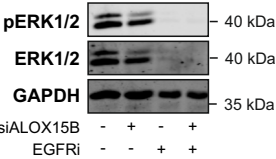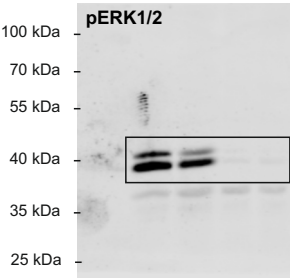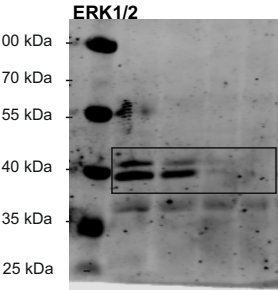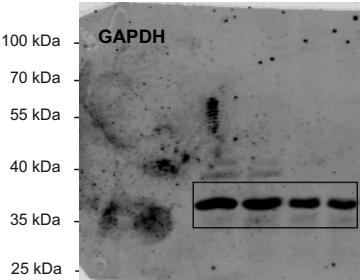

Fig.6B

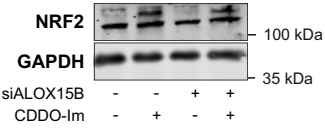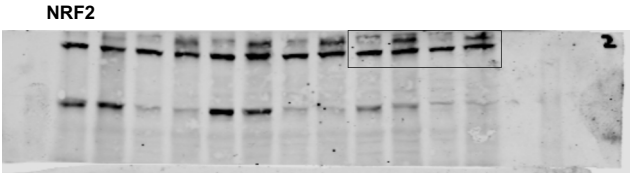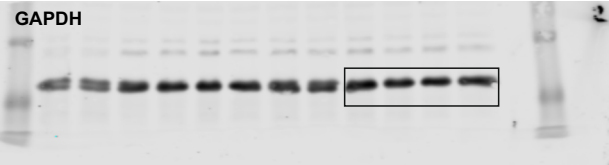

Fig.6D

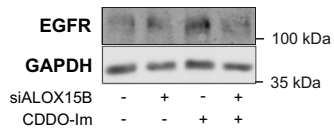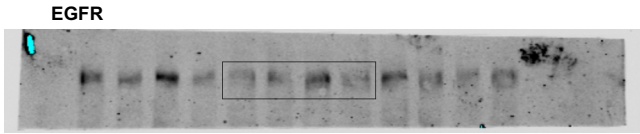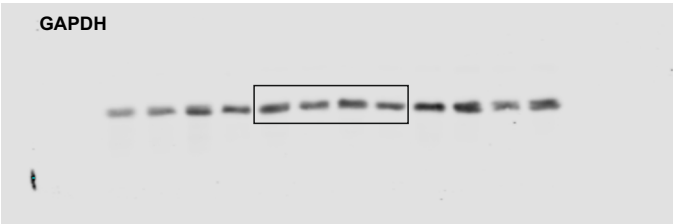

Fig.6E

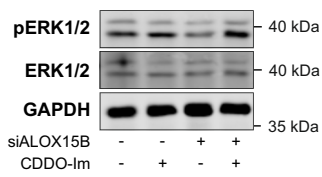

pERK1/2

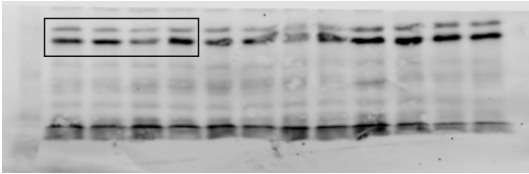

ERK1/2

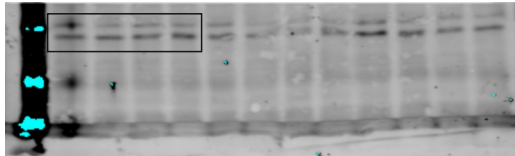

GAPDH

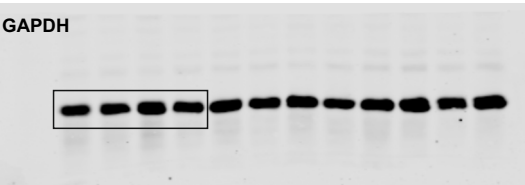

Fig.S1A

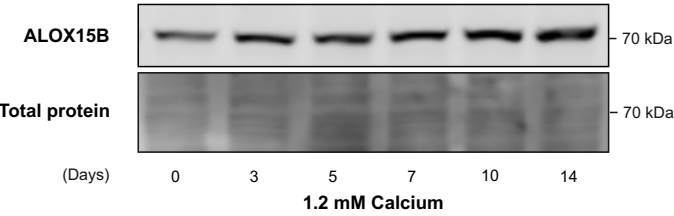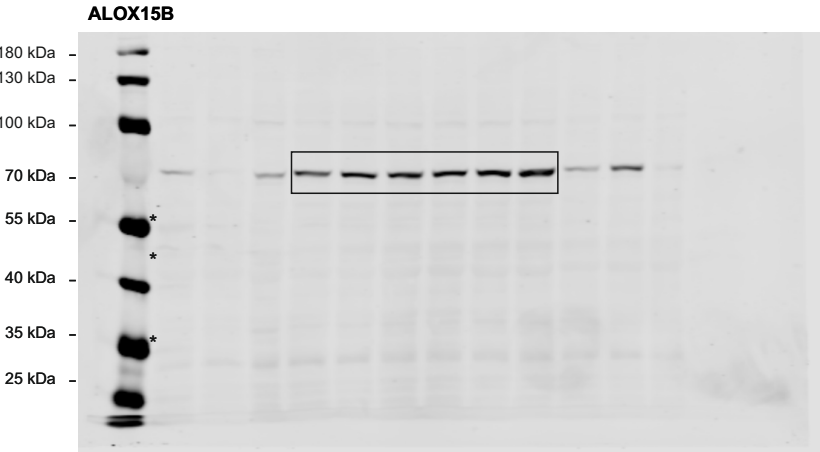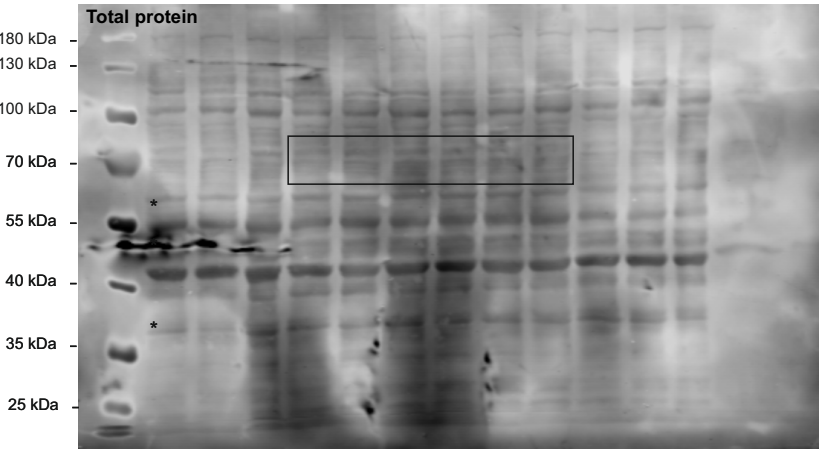

Fig.S3

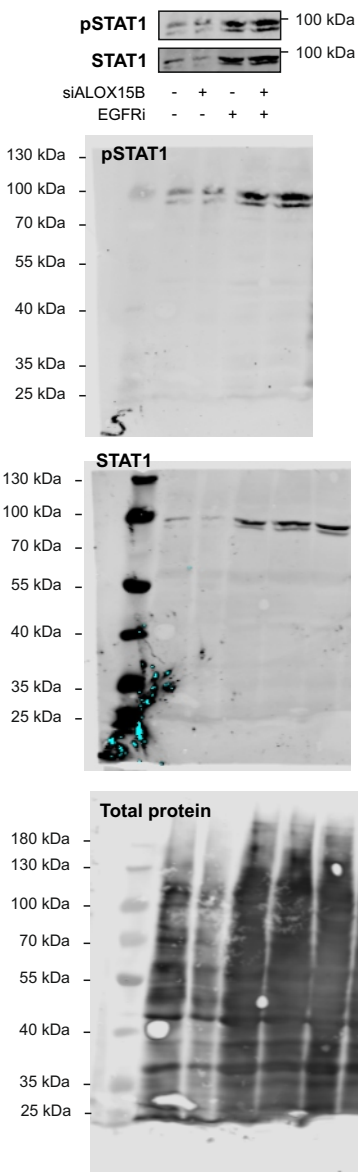

Fig.S4C

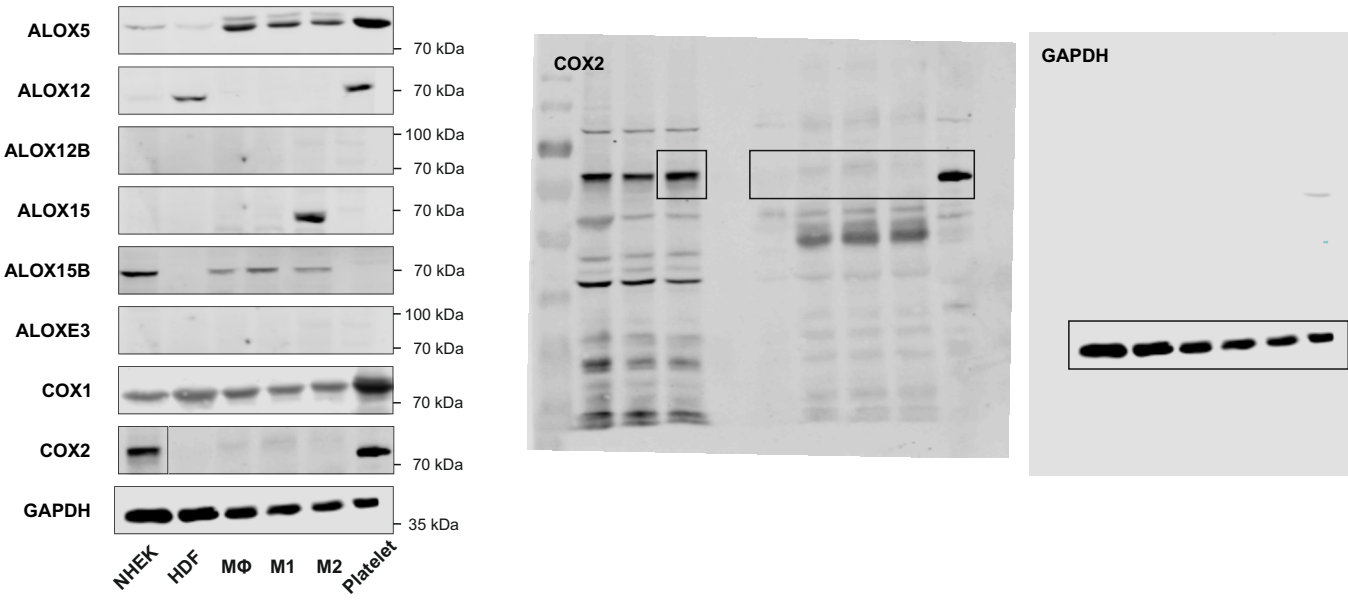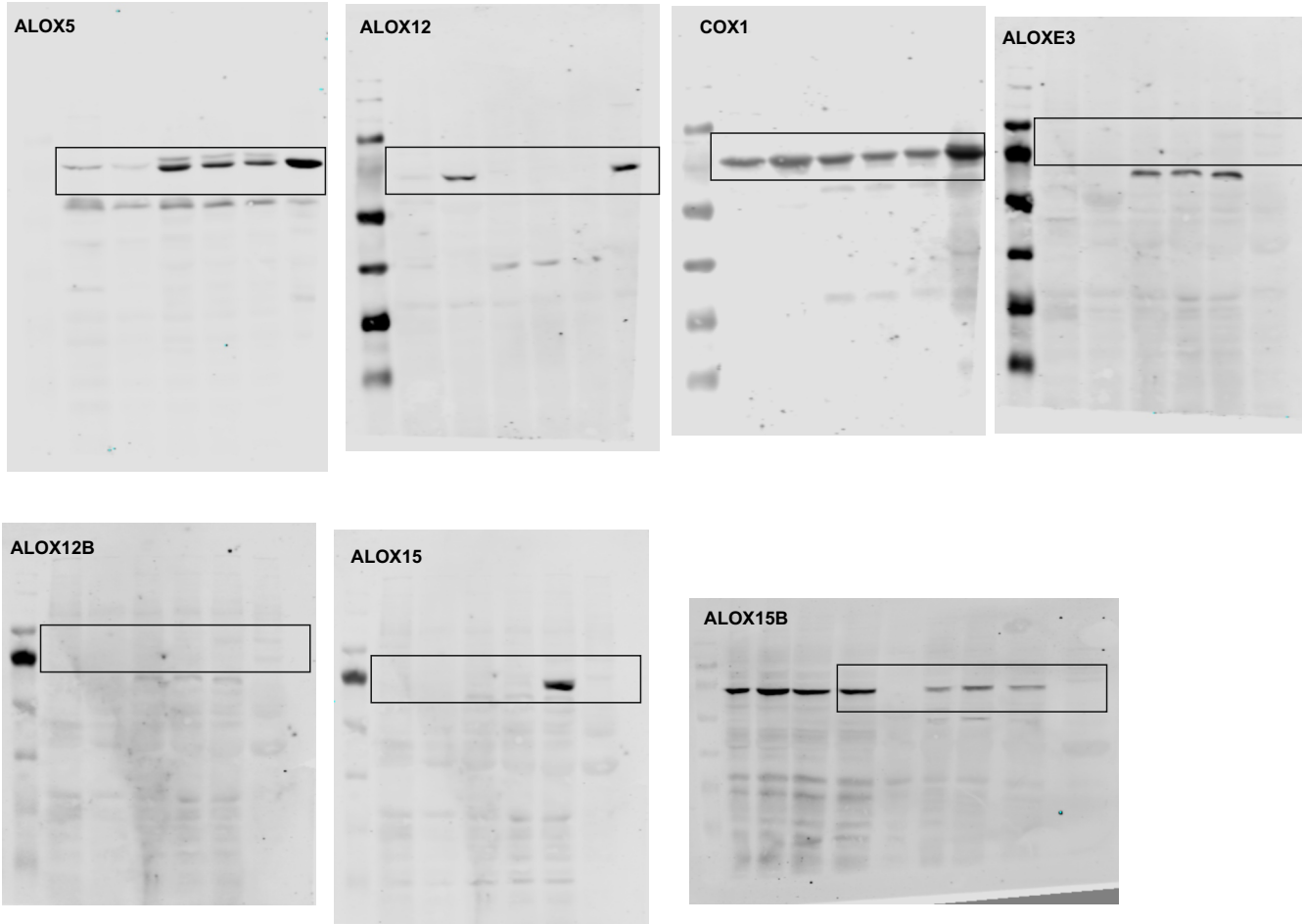

Supplement: Supplementary file 2 — Original Western Blots [file 41419_2025_7357_MOESM2_ESM.pdf]
